# GWASinspector: comprehensive quality control of genome-wide association study results

**DOI:** 10.1093/bioinformatics/btaa1084

**Published:** 2021-01-08

**Authors:** Alireza Ani, Peter J van der Most, Harold Snieder, Ahmad Vaez, Ilja M Nolte

**Affiliations:** Department of Epidemiology, University of Groningen, University Medical Center Groningen, 9700 RB Groningen, The Netherlands; Department of Bioinformatics, Isfahan University of Medical Sciences, 8174673461 Isfahan, Iran; Department of Epidemiology, University of Groningen, University Medical Center Groningen, 9700 RB Groningen, The Netherlands; Department of Epidemiology, University of Groningen, University Medical Center Groningen, 9700 RB Groningen, The Netherlands; Department of Epidemiology, University of Groningen, University Medical Center Groningen, 9700 RB Groningen, The Netherlands; Department of Bioinformatics, Isfahan University of Medical Sciences, 8174673461 Isfahan, Iran; Department of Epidemiology, University of Groningen, University Medical Center Groningen, 9700 RB Groningen, The Netherlands

## Abstract

**Summary:**

Quality control (QC) of genome wide association study (GWAS) result files has become increasingly difficult due to advances in genomic technology. The main challenges include continuous increases in the number of polymorphic genetic variants contained in recent GWASs and reference panels, the rising number of cohorts participating in a GWAS consortium, and inclusion of new variant types. Here, we present GWASinspector, a flexible R package for comprehensive QC of GWAS results. This package is compatible with recent imputation reference panels, handles insertion/deletion and multi-allelic variants, provides extensive QC reports and efficiently processes big data files. Reference panels covering three human genome builds (NCBI36, GRCh37 and GRCh38) are available. GWASinspector has a user friendly design and allows easy set-up of the QC pipeline through a configuration file. In addition to checking and reporting on individual files, it can be used in preparation of a meta-analysis by testing for systemic differences between studies and generating cleaned, harmonized GWAS files. Comparison with existing GWAS QC tools shows that the main advantages of GWASinspector are its ability to more effectively deal with insertion/deletion and multi-allelic variants and its relatively low memory use.

**Availability and implementation:**

Our package is available at The Comprehensive R Archive Network (CRAN): https://CRAN.R-project.org/package=GWASinspector. Reference datasets and a detailed tutorial can be found at the package website at http://gwasinspector.com/.

**Supplementary information:**

Supplementary data are available at *Bioinformatics* online.

## 1 Introduction

Recent genome-wide association studies (GWASs) use imputation reference panels based on next-generation sequencing technology. This has created a number of difficulties for quality control (QC) of the GWAS result files as a vital step of the analysis pipeline. Software packages like GWAStools (Gogarten *et al.*, 2012), GWAtoolbox (Fuchsberger *et al.*, 2012), QCGWAS (van der Most *et al.*, 2014) and EasyQC (Winkler *et al.*, 2014) have been previously developed for this purpose. However, these do not properly address current key challenges including diversity of allele frequency reference panels, inclusion of new variant types such as insertion/deletion (indel), and multi-allelic variants. Furthermore, the sheer data size of the result files as well as the reference panel(s) pose a problem. This issue is more evident in meta-analysis projects involving numerous result files from multiple sources, which warrants the need for a more time-efficient QC software. This motivated us to develop a new package for the QC of GWAS result files addressing the above mentioned shortcomings. GWASinspector is a feature-rich and easy-to-use package written in the R programming language. It evaluates GWAS result files and reports key QC metrics. Its ability to efficiently handle big data, indel and multi-allelic variants and to generate comprehensive graphic reports are the main strengths of this software package. Besides QC of single files, GWASinspector can be used in large-scale consortium projects to check for systematic differences between the reported results from different cohorts and generate cleaned, harmonized GWAS files ready for meta-analysis.

## 2 Implementation

**Fig 1.**
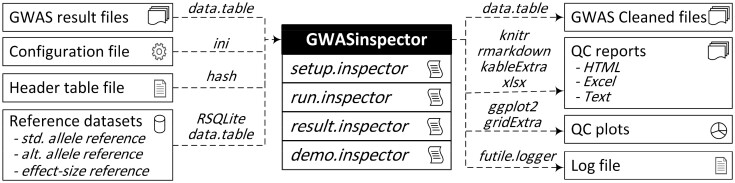
Components of GWASinspector. Contributing packages for each function are named on the dashed lines and are all available from the Comprehensive R Archive Network (https://cran.r-project.org). Abbreviations: std. = standard; alt. = alternate

GWASinspector is developed using S4 object models in R and is publicly available from the Comprehensive R Archive Network (CRAN). In addition, the website at http://GWASinspector.com provides reference databases alongside a detailed tutorial. It is designed to be friendly to use even for users with minimal programming background. All standard delimited text file formats, either raw or compressed as gzip files, are supported for analysis. User options and QC parameters are controlled through a configuration file. A sample configuration file is embedded in the package as an example. This file comes with full internal documentation in the form of comments and examples to make customization easy for novice users. A schematic view of the package is presented in Figure 1. More details on GWASinspector features, comparison with other packages, and sample QC reports are provided in the Supplementary Material. 

### 2.1 Methods

The validity of a GWAS result file can be compromised by accidental mix-up of columns, improper data merging, incorrect statistical analysis, duplicated records, missing data, variant imputation problems, study-level problems like population stratification or, in case of meta-analysis, inconsistency between participating studies. Thus, strict QC procedures are required. The first step includes checking the consistency and integrity of the files. Next, unusable data, including duplicated variants or variants that miss crucial information, are removed. The remaining data are then compared with the variant reference databases for allele and frequency matching, and (optionally) effect sizes are compared to previously published results. Harmonized marker IDs are generated using the combination of chromosome, position and type, for efficient variant matching with the reference datasets, and for handling multi-allelic and indel variants. GWASinspector will automatically generate (i) cleaned, harmonized GWAS files; and (ii) a variety of QC reports, statistics and plots, e.g. variant quality distribution plots, allele frequency correlation plots, Manhattan and QQ plots, genomic control reports, between-study comparison reports, etc. All important events are captured in a log file to monitor every step of the analysis process and to localize possible problems.

### 2.2 Reference datasets

GWASinspector comes with a variety of prepared reference datasets covering different human genome builds (NCBI36, GRCh37 and GRCh38), different resources (HapMap, 1000G, dbSNP, HRC, UK10K and TOPMED) and more importantly different variant types (multi-allelic and indel variants). These reference datasets are used to check alleles as well as allele frequencies to ensure they are all in the same configuration. We made use of the SQLite engine (https://www.sqlite.org) to generate the reference dataset because it is fast, reliable and portable across different platforms.

Similarly, previously published GWAS results can be used to generate variant effect-size reference datasets, in order to check the validity of the reported data. As a running example, effect-size reference datasets for heart rate variability (HRV) measures (Nolte *et al.*, 2017) and blood pressure (Evangelou *et al.*, 2018) were prepared via the data available from the GWAS catalogue (https://www.ebi.ac.uk/gwas/).

### 2.3 Output report files

A detailed report of the QC results is automatically saved as easy-to-read text, Excel and HTML files. The HTML version is the most complete report as it contains both QC summary report and plots in one organized portable file (see Supplementary Material for sample reports). In addition to separate reports for each GWAS file, a between-study comparison report is also created.

### 2.4 System requirements

GWASinspector is a cross-platform package with minor dependencies and can be run on a standard personal computer. However, to efficiently analyze a full-sized GWAS result file, a computer equipped with 64-bit operating system, Intel Core i7 CPU or equivalent, and at least 36 Gigabytes of RAM is recommended. Time estimate for inspection of a file containing approximately 20 million records, using a reference panel with approximately 80 million variants, is around 30 min on a high-performance computer (less if plots are skipped).

## 3 Usage

A demo function and sample data are available to explain the package and explore its features. A fast run on the first 1000 lines of a dataset can be done prior to full inspection, to check if it is correctly configured. This package has been successfully applied for the QC of approximately 500 GWAS result files coming from 23 cohorts in the second meta-analysis of the Genetic Variance in Heart Rate Variability (VgHRV) consortium (Nolte *et al.*, 2017).

## Supplementary Material

btaa1084_Supplementary_Data
