# Supplementary material for: GWASinspector: comprehensive quality control of genome-wide association study results
Source: Bioinformatics. 2021 Jan 8;37(1):129–30. doi: 10.1093/bioinformatics/btaa1084 (PMC8034536; doi:10.1093/bioinformatics/btaa1084)
Supplement: btaa1084_Supplementary_Data [file btaa1084_supplementary_data.zip › GWASinspector_features&comparison.pdf]

SUPPLEMENTAL MATERIAL

GWASinspector: comprehensive quality control of genome-wide association study results

Alireza Ani, Peter J. van der Most, Harold Snieder, Ahmad Vaez, Ilja M. Nolte

Table of Contents

GWASinspector package features 2

Comparison of GWASinspector package with other QC tools 4

- Methods 4
- Plotting 5
- Results 6
- EasyQC Package 13
- QCGWAS package 18
- GWAtoolbox package 19
- GWAStools package 20

Conclusion 20

Acknowledgement 20

References 21

GWASinspector package features

GWASinspector package is a simple, user-friendly application for thorough quality control of genome-wide association study (GWAS) result files. The main advantages of this package are:

1- **Easy to use:** GWASinspector is specifically developed for the purpose of a thorough quality control of the GWAS result files without the need to define extensive running parameters. Only the most essential settings such as paths, file loading/saving settings and thresholds are required.

2- **Optimized for large-scale meta-GWASs:** Pooling the results from many individual GWAS studies into a meta-analysis is a commonly used method to detect variants with low or modest effects. Consequently, a huge amount of data needs to be quality controlled and processed before any conclusions can be made. In addition to checking and reporting on individual result files, GWASinspector can be used in large-scale consortium projects for generating between-study comparison reports with essential quality control (QC) metrics and plots to check for systematic differences among the included studies. Besides the QC reports, an R object file stores raw QC metrics from each inspected result file. This data can later be used to compare GWAS result files from different pipelines together without the need to re-run the QC function.

3- **Generates cleaned, harmonized files:** besides checking the files and reporting the problems, GWASinspector automatically generates cleaned, harmonized files ready for meta-analysis.

4- **Broad range of allele reference datasets:** standard allele references covering different genome builds (NCBI36, GRCh37 and GRCh38) as well as different resources (HapMap, 1000G, dbSNP, HRC, UK10K and TOPMED) are available from our website (table 1).

Table 1. Allele reference datasets prepared for GWASinspector package

Reference dataset	Genome build	Number of variants
1000 Genomes Project *	GRCh37	84,346,970
dbSNP_GRCh37p13 *	GRCh37	82,798,854
dbSNP_GRCh38p7 *	GRCh38	82,285,539
HRC_r1-1	GRCh37	40,176,563
UK10K_COHORT_20160215 *	GRCh37	46,023,417
TOPMED *	GRCh37	162,739,927
HapMap_CEU_r28_b36	NCBI36	4,026,340

* Including multi-allelic and indel variants.

5- **Thorough matching of multi-allelic variants.**

6- **Thorough matching of indel variants.**

7- **Effect-size comparison:** an effect-size reference database can be used to test the concordance between the reported effect-sizes from a GWAS result file and a given dataset. This could be especially useful in a meta-analysis for examining the concordance between the reported effect sizes from different cohorts. In the case of a single GWAS result file inspection, a good correlation value between the obtained and previously-reported variant effect sizes could ensure the accuracy of the study and GWAS analysis.

8- **Automatic column name recognition:** Correct recognition of columns is very crucial for any software and in most of the analytical tools users are asked to define the name of the columns if they differ from the standard names known to the software. Contrary to that, GWASinspector uses a translation table file for this purpose which handles column recognition. Another advantage of this approach is that files with different column names can be easily inspected together, compared to the first method where column names and their package equivalent should be re-configured for each input file separately in the configuration script (if different from each other).

9- **Automatic generation of well-organized reports:** for the best user experience GWASinspector generates separate QC reports for each input result file in text, HTML and excel formats. Visual aspects of the reports are very appreciated in GWASinspector and extra effort was put in for utilizing competent report generator packages. Plotting was done via ggplot2 package instead of native R graphics for the same reason.

10- **Compatibility with other analytical applications:** Column names can be renamed automatically in the cleaned result files for compatibility with other analytical applications (e.g. META, GWAMA, PLINK and GenABEL).

11- **Application of S4 object system:** GWASinspector uses S4 object system in R to provide formally defined methods and classes.

12- **Indexed in CRAN:** GWASinspector is indexed in CRAN. So, package installation and update are simple and, in particular, the dependency packages will install automatically.

13- **Quick start guide:** we have developed a detailed tutorial on how to run a proper QC pipeline over GWAS result study files and then interpret the QC reports.

14- **Detailed logging of events.**

Comparison of GWASinspector package with other QC tools

Software packages like GWAStools (1), GWAtoolbox (2), QCGWAS (3) and EasyQC (4) have been previously developed for QC of GWAS result file. In this section, a comparison report covering main features and performance of these packages is provided. As each package has its own architecture with cons and pros, only the main differences observed in their QC pipeline are discussed.

GWAStools does not offer a QC pipeline and only some of the QC metrics such as distribution of variables (e.g. P-value), QQ plots, Manhattan plot, etc. are available through separate functions. Besides, it does not generate any specific QC reports. Therefore, GWAStools was not included in the following comparison tables.

The remaining four packages, i.e. GWASinspector, EasyQC, GWAtoolbox and QCGWAS, cover the basic needs for a quality control pipeline of GWAS result files. Checking the consistency of an input file, existence of the minimal required information, detection of unwanted variants (e.g. duplicated, incomplete variants), detection of unexpected values (e.g. out-of-range values or wrong data type), assessing the distribution of the main summary statistics and calculation of popular QC metrics are common among all.

The main differences in the QC pipeline of the abovementioned four packages are:

- Configuring the QC parameters
- Variant matching with an allele reference panel
- Inclusion of multi-allelic variants (SNP and indel) in the reference panel for variant matching
- Inclusion of indel alleles in the reference panel for variant matching
- Available reference panels for different human genome builds
- Comprehensiveness and format of the generated QC reports
- Required hardware/software resources

Methods

In order to compare the performance and computational requirements of these four packages, two sample compressed GWAS result files were analyzed in each package.

Specifications of the sample files used for testing the packages

#	Name	Dimensions (rows x columns)	Size
1	File1.txt.gz	12,148,301 x 14	382,588 KB
2	File2.txt.gz	12,148,372 x 14	382,601 KB

GWASinspector, EasyQC and QCGWAS packages were tested on the Peregrine High Performance Computing (HPC) cluster (5) and separate jobs were scheduled on one machine with the same configurations. Job submission was handled by Slurm Workload Manager (v.20.02.4). Memory usage and running times were taken from the server scheduler report.

- Operating system: CentOS Linux 7
- CPU: Intel Xeon 2.5 GHz
- Reserved RAM memory: 80GB
- R version 3.6.1 (2019-07-05)

GWAtoolbox requires a very old version of R (v.2) which was not available on Peregrine HPC. For this reason, an office server was set up and prepared for running the package. Variant harmonization was not tested because this functionality and the required VCF reference files are not explained in the package manual or website (please check below for more detail). Memory usage for GWAtoolbox was estimated from built-in Windows Task Manager tool. Running time was taken from the package output to R console after the job was terminated.

The configuration of the server for running GWAtoolbox is as follows:

- Operating system: Windows server 2016 x64
- CPU: Intel Xeon 2.2 GHz
- Available memory: 64GB
- R version 2.15.3 (2013-03-01)

Plotting

Plotting is a time-consuming task. Considering that different number of plots are generated in each package, the QC pipelines were tested twice:

1- Testing with the default number of figures

- All available plots were selected in GWASinspector and QCGWAS.
- For GWAtoolbox, verbosity level for graphical output was set to 2 (maximum).
- As the number and type of generated plots in EasyQC must be manually defined in the configuration script, the sample file (available from https://homepages.uni-regensburg.de/~wit59712/easyqc/1000g/fileqc_1000G.ecf) was used as a template.
- Some of the figures include multiple subplots; so the number of generated files and total number of plots are mentioned in the comparison table.

2- Testing with no figures

Similar QC tasks compared to the previous testing conditions were performed except for generation of the plots.

- Plotting was turned off in GWASinspector and QCGWAS packages, but all remaining tasks compared to the previous test were performed.
- No plots were defined in the EasyQC configuration file and AFCHECK step was also removed. The AFCHECK command, used for checking allele frequencies and strand orientation, was ignored because it automatically generates allele frequency scatterplots as an output which cannot be turned off. Thus, allele frequency correlation was not calculated and EasyQC did one less QC task compared to the previous test.
- Verbosity level for GWAtoolbox graphical output was set to 1 (minimum) for this test. Yet still the same number of plots were generated as when it was set to 2 (maximum).

Results

Basic QC tasks such as checking the consistency and content of the files, detection of problematic variants (e.g. duplicated, incomplete variants) or unexpected values (e.g. out-of-range values or wrong data type), and calculation of popular QC metrics were covered in all four pipelines. However, different methods are used for configuring the pipeline, QC process and generation of the reports. Table 2 provides an overview of the package specifications and features.

Contrary to GWASinspector, EasyQC and GWAtoolbox, which all use a configuration script for defining QC criteria, parameters in QCGWAS are passed to the main function as a long list. Regarding the QC steps, a pre-defined automatic pipeline is implemented in QCGWAS, GWAtoolbox and GWASinspector, but, this is not the case for EasyQC, and each QC task must be specified in the configuration file.

GWASinspector utilizes the S4 object oriented programming model in R. Items from the configuration file are read first and an object of the Inspector class will be instantiated if the parameters are valid. This object can then be viewed or even changed in R environment before starting the pipeline. Also, a fast-run is available to check whether all parameters are set correctly.

Regarding the allele frequency reference panels, QCGWAS only has the data for NCBI36 (HapMap project) and EasyQC provides the data from NCBI36 and GRCh37 (HapMap, 1000G and HRC projects). GWASinspector covers NCBI36, GRCh37 and GRCh38 and reference data from the 1000G, UK10k, TOPMED, dbSNP, HRC and HapMap projects are available. Moreover, thorough allele

matching for multi-allelic and indel variants is only available in GWASinspector. EasyQC does allow for indel matching, but alleles are abbreviated to I/D in the reference files.

QC reports are another important aspect of the comparison between the packages. GWASinspector generates both comprehensive file-specific QC reports and between-study comparison reports in text, HTML and Excel formats. On the other hand, tab/comma delimited text files are used in other packages. GWAtoolbox also generates HTML files, but the between-study comparison report only includes the effect-size box plots without any further data.

The benchmark report in table 3 provides an analysis of the performance from running two sample GWAS result files in each one of the four packages. Between these packages, GWAtoolbox has the fastest pipeline and also requires the least memory, but this could be attributed to absence of the variant matching process. QCGWAS was in the second place, however, considering that the only available reference data for this package comes from the much smaller NCBI36 reference panel, this result does not correctly indicate a better performance when compared with EasyQC and GWASinspector. QCGWAS uses `read.table()` and `write.table()` functions which are inefficient and very time-consuming for working with current GWAS result files. Moreover, the reference file is saved as an R object file (.RData extension) and requires loading with the `load()` function at first.

In comparison with QCGWAS, loading of the results and final saving of the files are faster in EasyQC. EasyQC and GWASinspector are compatible with up-to-date reference panels (e.g. GRCh37 human genome build), but different methods are selected for handling such data. The reference data for EasyQC are stored as tab delimited text files. Multiple files are required for population specific data and the whole file should be loaded for variant matching which is time and memory-consuming. For this reason, variant matching process in GWASinspector is based on the SQLite (6) technology. In this approach, multiple populations are included in a single database and SQL syntax query is used to search the required variants without the need to load the whole data. In addition, GWASinspector utilizes the `data.table` package (7) which is specifically developed for efficient loading and aggregation of large data files and works much faster than native R libraries (e.g. `R.utils`).

The below benchmark reports compare the estimated required times for 1) loading and 2) writing a sample GWAS result file and 3) loading time for a sample reference panel between different approaches used in QCGWAS, GWASinspector and EasyQC packages.

The following table compares the loading time of a sample gzipped GWAS result file (12,148,301 rows x 14 columns) between the three compared packages.

Package	Time
data.table::fread (GWASinspector) #	45.27 seconds
utils::read.table (QCGWAS) #	530.26 seconds
EasyQC *	100.50 seconds

# microbenchmark package was used for estimating the times.
* Required time was taken from the EasyQC output file.

The benchmark report below indicates the writing time of a sample gzipped result file (~12,000,000 rows x 14 columns).

Package	Time
data.table::fwrite (GWASinspector) #	39.72 seconds
utils::write.table (QCGWAS) #	486.20 seconds
EasyQC *	163.80 seconds

# microbenchmark package was used for estimating the times.
* Required time was taken from the EasyQC output file.

Finally, the benchmark report below indicates loading time for a sample reference file (e.g. 1000G reference panel with 84,400,371 rows and 4 columns) in different file formats for each package.

Package	Time
SQLite database (GWASinspector)*	NA
R object (QCGWAS) **	310.26 seconds
Tab delimited file (EasyQC)#	187.20 seconds

* database technology is used which does not require loading.
**base::load() method is used. microbenchmark package was used for estimating the time.
Required time was taken from the EasyQC report file.

It should be noted that the provided times are estimates from our assessment and might differ a bit if tested elsewhere. However, they demonstrate that GWASinspector works relatively faster and requires relatively lower memory resources when compared to the EasyQC package under the same conditions.

A brief introduction for each package and important differences compared to GWASinspector are given in the following section.

Table 2. Comparison table describing the difference between GWASinspector, EasyQC, GWAtoolbox and QCGWAS

	GWASinspector	EasyQC	GWAtoolbox	QCGWAS
**Programming language**	R	R	R<3.0.0, C++	R
**R object system**	S4	?	?	S3
**Websites**	http://gwasinspector.com	https://www.uni-regensburg.de/medizin/epidemiologie-praeventivmedizin/genetische-epidemiologie/software	http://www.eurac.edu/en/research/health/biomed/services/Pages/GWAtoolbox.aspx	https://cran.r-project.org/web/packages/QCGWAS/index.html
**Available from CRAN or Bioconductor**	CRAN	None	None	CRAN
**Version**	1.4.8 (2020-07-28)	23.8 (2020-08-14)	2.2.4-7 (2013-07-30)	1.0-8 (2014-02-12)
**Quality control pipeline**	Pre-defined automatic pipeline is implemented.	QC tasks must be specified in the configuration file.	Pre-defined automatic pipeline is implemented.	Pre-defined automatic pipeline is implemented.
**Loading the input files**				
**Setting the input files**	Filenames are selected based on a specified pattern.	Filenames must be specified individually.	Filenames must be specified individually.	Filenames must be specified individually.
**Column identification**	Translation table is used.	Should be set in the configuration script.	Should be set in the configuration script.	Translation table is used.
**Reference panels**				
**Available allele reference databases**	1000G (GRCh37)<br>dbSNP (GRCh37,38)<br>TOPMED (GRCh37)<br>UK10K (GRCh37)<br>HRC (GRCh37)<br>HapMap (NCBI36)	1000G (GRCh37)<br>HRC (GRCh37)<br>HapMap (NCBI36)	None	HapMap (NCBI36)
**Allele matching for multi-allelic variants**	Yes (GRCh37- GRCh38)	No	Data not available	No

	GWASinspector	EasyQC	GWAtoolbox	QCGWAS
**Allele matching for insertions or deletions**	Yes (GRCh37- GRCh38)	No	Data not available	No
**QC Reports**				
**Sample files containing duplicated variants**	Yes	Yes	No, included in the report file	Yes
**Sample files containing mismatched variants**	Yes, separate files for bi-allelic and multi-allelic variants	Yes	No	Yes
**Sample files containing problematic variants**	Yes	Yes	No	Yes
**Report files**	Individual files; and between-file comparison report:<br>• text files (1 for each input file, 1 for all)<br>• HTML files (1 for each input file, 1 for all)<br>• Excel file (1 sheet for each input file, 1 sheet for all)<br>• Log file	• One tab delimited text file merging QC metrics for all input result files<br>• Log file	Separate reports for each file:<br>• Text report<br>• HTML file<br>• CSV file, including variants distribution and QC metrics<br>Between-file comparison report:<br>• HTML file (only contains effect-size boxplots)	Individual files; and between-file comparison report in text format.

Table 3. Benchmark report from running GWASinspector, EasyQC, GWAtoolbox and QCGWAS

	**GWASinspector**	**EasyQC**	**GWAtoolbox**	**QCGWAS**
**Allele reference file**	1000GENOMES-phase_3_EUR.sqlite	1000GP_p3v5_legends_rbind.noDup.noMono.noCnv.noCnAll.afref.ALL.txt.gz	None	HapMap_CEU_r28_b36_EDIT_v10c.RData
**Number of variants in the allele reference file**	84,346,970	84,400,372	-	4,026,340
**Size of the allele reference file (compressed/decompressed)**	1.16G/4.11G	415M/1.9G	-	35.4M
**Testing with figures**				
**Maximum memory used**	18.66G	27.29G	1.6G	9.50G
**Used wall time**	00:55:01*	01:12:48	00:18:22	00:35:47
**Used CPU time**	00:54:24	01:12:23	-	00:35:44
**Generated plots**	Study-specific plots<br>• Allele frequency correlation scatter plots (3 subplots)<br>• P-value correlation scatter plot<br>• QQ plots (4 subplots)<br>• Histograms (6 subplots)<br>• Effect-size correlation scatterplot<br>• Manhattan plot<br><br>Between-file comparison plots<br>• Skewness-kurtosis plot<br>• Precision plot<br>• Effect-sizes box plots	• Allele frequency correlation scatterplot<br>• P-value correlation scatter plot (PZLPOT)<br>• QQPLOT<br>• RPLOT	Study-specific plots<br>• P-value histogram plots<br>• QQplots (x3)<br>• Effect-size boxplots (3 subplots)<br>• Beta distribution plots (2 subplots)<br>• Column specific distribution plots (e.g. effect-size, N, SE, etc., 3 subplots)<br><br>Between-study comparison plots<br>Effect-sizes box plots (x2)	Study-specific plots<br>• Allele frequency correlation scatter plots (x3)<br>• P-value correlation scatter plot<br>• QQ plots (4 subplots)<br>• Histograms (6 subplots)<br>• Manhattan plot<br><br>Between-study comparison plots<br>• Skewness-kurtosis plot<br>• Precision plot<br>• Effect-sizes box plots
**Number of figure files/plots**	15 files – 28 plots	6 files – 8 plots	22 files – 36 plots	17 files – 29 plots

	GWASinspector	EasyQC	GWAtoolbox	QCGWAS
**Testing without figures**				
**Maximum memory used**	17.40G	27.71G	1.6G	8.93G
**Used wall time**	00:40:23	00:56:58	00:17:49	00:25:03
**Used CPU time**	00:39:52	00:56:41	-	00:24:59
**Generated plots**	None	None	The same as previous test.**	None

Abbreviations: G, gigabytes; M, megabytes
* Time is presented as HH:MM:SS
** verbosity level for graphical output was set to 1 (minimum), but the same number of figures were generated as the time when verbosity level was set to 2 (maximum).

EasyQC Package

EasyQC is an advanced R package for QC of GWAS result files (4). Version 23.8 of this package was very recently released (2020-08-14), six years after the previous version (version 9.2, published 2014-09-24) (8). The package manual for the latest version was not yet available at the time of preparing this report, so the previous manual (v9.2) was used.

Below are the most important differences between the QC pipeline from GWASinspector and EasyQC:

- Prepared allele reference datasets for GWASinspector package cover more reference panels and genome build versions. Also, the 1000Genome project study reference file has more variant detail in GWASinspector compared to EasyQC. Specifically, 1) it appears that multi-allelic variants are either missing from the EasyQC reference files or only one of the forms is present 2) indel alleles are shrunk to I/D.

  Below table shows the files for CEU HapMap imputed data, 1000G phase1 version3, 1000G phase3 version5 and HRC reference data, available from the EasyQC package homepage (8).

Table 4. Allele reference datasets prepared for EasyQC package.

File name	Variant count
allelefreq.1000G_EUR_p1v3.impute_legends.noDup.noX.v2.gz*	38,043,496
1000GP_p3v5_legends_rbind.noDup.noMono.noCnv.noCnAll.afref.EUR.txt.gz*	24,848,280
1000GP_p3v5_legends_rbind.noDup.noMono.noCnv.noCnAll.afref.ALL.txt.gz	84,400,371
HRC.r1-1.GRCh37.wgs.mac5.sites.tab.cptid.maf001.gz	16,430,621
AlleleFreq_HapMap_CEU.v2.txt.gz	2,532,578

* Separate reference files for EAS, SAS, AMR, AFR population are also provided. Available from: https://www.uni-regensburg.de/medizin/epidemiologie-praeventivmedizin/genetische-epidemiologie/software/

As an example, EasyQC's "allelefreq.1000G_EUR_p1v3.impute_legends.noDup.noX.gz" reference file has the data for European population from the 1000G study (phase1 version3). It has 38,043,496 rows without any duplicated ids (cptid) and a sample row is as follows:

cptid	a0	a1	eaf
10:100000012	G	A	1

cptid: <CHR>:<POSITION><TYPE>' with TYPE being ':ID' for INDELs or blank '' for SNPs, a0: effect allele, a1: other allele; eaf: effect allele frequency

Distribution of the alleles (a0 and a1) in this file is as follows:

a0	N
G	10,691,309
A	7,645,153
T	7,634,713
C	10,677,817
D	551,762
I	842,742
Total	38,043,496

a1	N
A	10,396,425
G	7,959,680
C	7,946,261
T	10,346,626
I	551,762
D	842,742
Total	38,043,496

As another example, "1000GP_p3v5_legends_rbind.noDup.noMono.noCnv.noCnAll.afref.ALL.txt.gz" has the allele frequencies from the total 1000G study (phase3 version5). This file has a larger number of variants compared to the other population specific files from this reference. It has 84,400,371 rows without any duplicated ids (cptid) and sample rows are as follows:

cptid	ea	oa	eaf
10:100000003	C	T	0.999401
10:100024611:ID	D	I	0.730431
10:100019689:ID	I	D	0.997804

cptid: <CHR>:<POSITION><TYPE>' with TYPE being ':ID' for INDELs or blank '' for SNPs, ea: effect allele, oa:other allele; eaf: effect allele frequency

Distribution of the alleles (ea and oa) in this file is as follows:

ea	N
C	23,597,119
G	23,601,677
A	16,953,344
T	16,912,033
D	1,231,097
I	2,105,101
Total	84,400,371

oa	N
T	22,809,327
A	22,983,256
G	17,664,337
C	17,607,253
I	1,231,097
D	2,105,101
Total	84,400,371

Based on these allele distribution tables and lack of any duplicated variant IDs (cptid), it appears that multi-allelic variants are either missing from the abovementioned files or only one of the forms is present. While working with the EasyQC package as part of the comparison of GWASinspector with existing GWAS QC tools we encountered two major issues:

1- Correct multi-allelic variants were tagged as either "mismatch" or "duplicated" because the reference file lacked the other form of the variant or because both forms of a variant were present in the result file, but the reference file only included one of the forms.

For example, the below multi-allelic variant from a sample GWAS result file was considered a mismatch because the reference file only contains one of the possible forms:

CHR	POS	EFFECT_ALLELE	OTHER_ALLELE	N	EAF	SE	PVAL	HWE_P	BETA
8	34158826	T	G	2489	0.012	0.08265	0.2362	1	-0.09793

This is the variant in the EasyQC reference file:

Cptid	ea	oa	eaf
8:34158826	G	A	0.996206

And this is the variant data from the GWASinspector reference file:

CHR	POS	REF	ALT	AF
8	34158826	G	A,T	0.0037,0.0095

As another example, below variants from a sample GWAS result file were considered duplicates because both forms of the variants were present in the result file, but the reference file only included one of the forms:

CHR	POSITION	EFFECT_ALLELE	OTHER_ALLELE	EAF	SE	PVAL	HWE_P	BETA
1	100004209	G	GTTTT	0.133	0.0200	0.4304	0.5416	-0.0158
1	100004209	G	GTTTTT	0.132	0.0196	0.9946	0.9300	-0.0001
1	1000156	G	C	0.004	0.382	0.9836	1	-0.0078
1	1000156	T	C	0.725	0.0150	0.1322	0.9599	-0.0226

This is the variant data from EasyQC reference file:

cptid	ea	oa	eaf
1:100004209:ID	D	I	0.86861
1:1000156	C	G	0.960463

And this the variant data from GWASinspector reference file:

CHR	POS	REF	ALT	AF
1	100004209	G	GTTTT,GTTTTT,GTTTTTTT	0.0895,0.1571,0
1	1000156	C	G,T	0.002,0.7425

2- Mismatched indels (e.g. A/AT vs A/ATT) were overlooked and multi-allelic indels (e.g. A/AT vs A/AT,ATT,ATTT) could not be correctly matched. The reason for these issues is that reference files abbreviate the alleles to I/D instead of full allele information for the indel variants.

For example, this is the information in the GWASinspector reference file:

**CHR**	**POS**	**REF**	**ALT**	**AF**
1	10094536	T	TA,TAA	0.0209, 0.0716

And this is for the same variant in EasyQC reference file:

**Cptid**	**Ea**	**oa**	**eaf**
1:10094536:ID	D	I	0.926318

To resolve the above-mentioned issues, reference files for GWASinspector store actual alleles for indel variants and different forms of a multi-allelic variant (SNP and indel). As an example from GWASinspector, the "1000GENOMES-phase_3_EUR.sqlite" database file has the data for European population from the 1000G study (phase3 version5). The table below displays sample rows from this database.

#	REF	ALT	EUR_AF	hID
1	A	AC	0.4056	1:10177:2
2	C	G	0.0885	1:11008:1
3	T	A,G	0.3499,0.3857	3:191063:1
4	C	CT,CTCCTTCC,CTCCTTCCT,CTTCCT	0.1133,0,0.0646,0.0119	3:351075:2

This reference file stores (i) actual alleles for indel variants (numbers 1 and 4) and (ii) different forms of a multi-allelic variant for correct matching (numbers 3 and 4). In total, 5,665,373 multi-allelic variants are included in this specific database.

- All the input files must be separately declared in the EasyQC configuration script for analysis. On the other hand, GWASinspector uses pattern matching with regular expression compatibility for loading the files. Files from the input folder will be selected if their name match the pattern. For example, the below commands are required for loading 5 files in EasyQC:

```
EASYIN  --fileIn /path2input/GWAS1000G.STUDY1.file1.txt.gz
```

```
EASYIN  --fileIn /path2input/GWAS1000G.STUDY1.file2.txt.gz
EASYIN  --fileIn /path2input/GWAS1000G.STUDY1.file3.txt.gz
EASYIN  --fileIn /path2input/GWAS1000G.STUDY1.file4.txt.gz
EASYIN  --fileIn /path2input/GWAS1000G.STUDY1.file5.txt.gz
```

Equivalent settings in GWASinspector is:

```
dir_data = /path2input
filename = [1-5].txt.gz
```

- As mentioned previously, only the most essential settings such as paths, file loading/saving settings and study dependent thresholds (e.g. filtering high-quality variants, etc.) are required in GWASinspector. Steps such as removing the duplicated lines of data, removing variants with invalid/missing crucial values (e.g. alleles, beta, SE), matching the variants with allele reference data, calculation of descriptive statistics or generating comparative plots are elemental parts of a QC pipeline and will always be performed.

  In contrast, each QC task must be specified in the EasyQC configuration file. Although the possibility of defining parameters for each and every step gives more control to the users, this is an error-prone method and complicates matters. Considering that a QC pipeline has a specific routine in most cases, there is no need to outline all the basic steps. For example, the user must include below lines (selected from a long list of available parameters) in EasyQC configuration script for some of the abovementioned tasks and reports. Additional parameter lines and numerous plotting options are truncated:

```
CLEAN --rcdClean is.na(EFFECT_ALLELE)&is.na(OTHER_ALLELE) --
strCleanName numDrop_Missing_Alleles
CLEAN --rcdClean is.na(PVAL) --strCleanName numDrop_Missing_P
CLEAN --rcdClean is.na(BETA) --strCleanName numDrop_Missing_BETA
CLEAN --rcdClean is.na(SE) --strCleanName numDrop_Missing_SE
CLEAN --rcdClean is.na(EAF) --strCleanName numDrop_Missing_EAF
CLEAN --rcdClean is.na(N) --strCleanName numDrop_Missing_N
CLEAN --rcdClean is.na(IMPUTATION) --strCleanName
numDrop_Missing_Imputation
CLEAN --rcdClean PVAL<0|PVAL>1 --strCleanName numDrop_invalid_PVAL
CLEAN --rcdClean SE<=0|SE==Inf --strCleanName numDrop_invalid_SE
CLEAN --rcdClean EAF<0|EAF>1 --strCleanName numDrop_invalid_EAF
CLEAN --rcdClean IMPUTATION<0 --strCleanName
numDrop_invalid_IMPUTATION
EVALSTAT --colStat P
EVALSTAT --colStat BETA
EVALSTAT --colStat EAF
EVALSTAT --colStat HWE_P
EVALSTAT --colStat SE
MERGE ...
QQPLOT ...
RPLOT ...
SPLOT ...
AFCHECK ...
```

- EasyQC generates one tab delimited text report by merging QC metrics from all input files, which is hard to read in the original format. On the other hand, GWASinspector generates separate reports for file-level and between-study checks in various formats.

QCGWAS package

QCGWAS is an R package for QC of GWAS result files and is available from CRAN repository (3). The most current version of this package is 1.0-8 (published 2014-02-12) which was used for this report.

Below issues are the most important factors when comparing GWASinspector with QCGWAS:

- The latest available allele reference file for this package is from HapMap project (NCBI36) and is not further updated.
- This package does not utilize a configuration script and all QC parameters must be passed to the main function. Most of the parameters have default values and are not required, but considering the high number of available function parameters this can be confusing for the user.

  For example, the below command could be used for running the QC pipeline on two result files.

```
QC_series(
        data_files= c("data1.txt","data2.txt"),
        output_filenames = c("output1.txt","output2.txt"),
        dir_data = "C:/data/preQC",
        dir_output = "C:/data/postQC",
        dir_references = "C:/data/QC_files",
        save_final_dataset = TRUE,
        spreadsheet_friendly_log = TRUE,
        header_translations = “alt_headers.txt”,
        allele_ref_std =”ref.rdata”,
        na.strings = c("NA", "nan", "NaN", "."),
        imputed_T = c("1", "TRUE", "T", "yes", "YES", "y", "Y"),
        imputed_F = c("0", "FALSE", "F", "no", "NO", "n", "N"),
        imputed_NA = c(NA, "-"),
        order_columns = TRUE,
        out_header = "old",
        remove_X = FALSE,
        remove_Y = TRUE,
        remove_XY = TRUE,
        remove_M = TRUE,
        calculate_missing_p = FALSE,
        make_plots = TRUE,
        only_plot_if_threshold = FALSE,
        threshold_allele_freq = 0.95,
        threshold_p_correlation = 0.99,
        plot_cutoff_p = 0.05,
        allele_name_std = "HapMap",
        allele_ref_alt = "ref_other.txt",
        allele_name_alt = "Other",
        update_alt = FALSE,
        update_as_rdata = FALSE,
        threshold_diffEAF = 0.15,
```

```
use_threshold = 100000,
HQfilter_FRQ = 0.01,
HQfilter_HWE = 10^-6,
HQfilter_cal = 0.95,
HQfilter_imp = 0.3,
QQfilter_FRQ = c(NA, 0.01, 0.03, 0.05, 3),
QQfilter_HWE = c(NA, 10^-6, 10^-4),
QQfilter_cal = c(NA, 0.95, 0.98, 0.99),
QQfilter_imp = c(NA, 0.3, 0.5, 0.7, 0.9),
NAfilter = TRUE,
ignore_impstatus = FALSE,
minimal_impQ_value = -0.5,
maximal_impQ_value = 1.5,
plot_effectsizes = TRUE,
lim_effectsizes = c(-0.6, 0.6),
save_filtersettings = TRUE)
```

- QCGWAS generates study-level and between-study comparison report files. But, the reported QC metrics are in tab-delimited text format which is not easy on the eye.
- Reading and writing of large GWAS result files is slow due to using utils::read.table() , write.table() functions. In GWASinspector, these functions are replaced by the much faster data.table::fread() and fwrite() functions.

GWAtoolbox package

GWAtoolbox is an R-package for fast quality control and handling of data files obtained from multiple GWAS studies (2). This package was removed from the CRAN repository and the latest version (V.2.2.4-7, published 2013-07-30) is only available from the package website (9). It requires R v.2, which is outdated. Considering that R v3.0.0 was introduced in 2013, this package is not compatible with most of the modern computer clusters.

In the package functions list, `harmonize()` is described as a function for harmonizing SNP identifiers and alleles in the input file according to the provided VCF reference. However, there is no further information regarding this function or the required VCF references in GWAtoolbox manual or website.

Below issues are the most important factors when comparing GWASinspector with GWAtoolbox:

- This package is outdated and cannot be run on most of the modern computer clusters.
- Variant harmonization with an allele reference panel is in question.
- Similar to EasyQC package, column names and all the input files should be declared in the configuration script.
- Between-study comparison report (in HTML format) only contains effect-size boxplots with no further details.

GWAStools package

GWASTools (v.1.34.0) is an R package for performing GWAS studies (1) and is available from Bioconductor website (10). It is geared towards quality control, data cleaning, and analysis of GWAS data, and the vast majority of the papers that cited GWASTools used this package to perform a GWAS (11).

Below issues are the most important factors when comparing GWASinspector with QC features of GWAStools:

- Variant matching and harmonization with a standard allele reference is not available in GWAStools.
- GWAStools requires a specific data format. So, an additional data conversion step may be required to be able to use the package.
- Some QC metrics, such as distribution of variables (e.g. P-value), QQ plots, Manhattan plot, etc. can be generated separately through various package functions. But, unlike GWASinspector, GWAStools does not offer a QC pipeline for GWAS result files. Besides, it does not generate any specific QC reports.

Conclusion

The main advantages of GWASinspector are that (i) it is easy to use, (ii) it is able to efficiently and effectively deal with most recent human genome builds and new type of variants like insertion/deletion and multi-allelic variants, (iii) it requires relatively low memory resources and (iv) it runs relatively fast.

Acknowledgement

We would like to thank the Center for Information Technology of the University of Groningen for their support and for providing access to the Peregrine high performance computing cluster.

References

1. Gogarten SM, Bhangale T, Conomos MP, Laurie CA, McHugh CP, Painter I, et al. GWASTools: an R/Bioconductor package for quality control and analysis of genome-wide association studies. Bioinformatics. 2012 Dec 1;28(24):3329–31.

2. Fuchsberger C, Taliun D, Pramstaller PP, Pattaro C. GWAtoolbox: an R package for fast quality control and handling of genome-wide association studies meta-analysis data. Bioinformatics. 2012 Feb 1;28(3):444–5.

3. van der Most PJ, Vaez A, Prins BP, Munoz ML, Snieder H, Alizadeh BZ, et al. QCGWAS: A flexible R package for automated quality control of genome-wide association results. Bioinformatics. 2014 Apr 15;30(8):1185–6.

4. Winkler TW, Day FR, Croteau-Chonka DC, Wood AR, Locke AE, Mägi R, et al. Quality control and conduct of genome-wide association meta-analyses. Nat Protoc. 2014 May;9(5):1192–212.

5. Peregrine HPC cluster [Internet]. University of Groningen. 2015 [cited 2020 Aug 31]. Available from: https://www.rug.nl/society-business/centre-for-information-technology/research/services/hpc/facilities/peregrine-hpc-cluster

6. SQLite Home Page [Internet]. [cited 2020 Oct 4]. Available from: https://www.sqlite.org/index.html

7. Dowle M, Srinivasan A, Gorecki J, Chirico M, Stetsenko P, Short T, et al. data.table: Extension of "data.frame" [Internet]. 2020 [cited 2020 Oct 4]. Available from: https://CRAN.R-project.org/package=data.table

8. Software - Universität Regensburg [Internet]. [cited 2020 Aug 24]. Available from: https://www.uni-regensburg.de/medizin/epidemiologie-praeventivmedizin/genetische-epidemiologie/software/

9. Services GWAtoolbox [Internet]. [cited 2020 Aug 17]. Available from: http://www.eurac.edu/en/research/health/biomed/services/Pages/GWAtoolbox.aspx

10. Gogarten SM, Laurie C, Bhangale T, Conomos MP, Laurie C, Lawrence M, et al. GWASTools: Tools for Genome Wide Association Studies [Internet]. Bioconductor version: Release (3.11); 2020 [cited 2020 Aug 24]. Available from: https://bioconductor.org/packages/GWASTools/

11. RPubs - GWASTools Overview [Internet]. [cited 2020 Aug 17]. Available from: https://rpubs.com/karlkeat/gwastools
