# Supplementary material for: GWASinspector: comprehensive quality control of genome-wide association study results
Source: Bioinformatics. 2021 Jan 8;37(1):129–30. doi: 10.1093/bioinformatics/btaa1084 (PMC8034536; doi:10.1093/bioinformatics/btaa1084)
Supplement: btaa1084_Supplementary_Data [file btaa1084_supplementary_data.zip › Introduction_to_GWASinspector_v1.4.8.pdf]

Introduction to “GWASinspector”

This tutorial introduces the “GWASinspector” package, its general form and how to apply the algorithm on GWAS result files.

Developed by: Alireza Ani, Peter J. van der Most, Ahmad Vaez, Ilja M. Nolte

Version: 1.4.8

Date: September 2020

Contents

The GWASinspector package 3
Introduction 3
How to install GWASinspector 3
Package dependency list 3
Additional modules 4
Auxiliary files 5
Available functions 8
Output file naming conventions 12
Setting QC parameters in configuration file 15
Configuration file sections and properties 16
Selecting input names using the filename key 20
Step-by-step guide to run a QC 21
Step 1: Load the package 21
Step 2: Check R environment 21
Step 3: Download the standard allele frequency reference datasets 22
Step 4: Get the header-translation table 22
Step 5: Get the configuration file 22
Step 6: Modify the parameters in the configuration file 22
Step 7: Run the QC function 23
Quality control steps of GWAS result files 24
Phase 1: Loading the dataset 25
Phase 2: Data integrity & SNP check 26
Phase 3: Aligning alleles with reference and comparing the allele frequencies 28
Phase 4: Quality control of the other statistics 31
Phase 5: Generating the report 32
Phase 6: Saving the cleaned dataset 32
Phase 7: Comparing multiple GWAS results files 32
Interpreting the QC reports 33
The overall report 33
The individual file report 36

The GWASinspector package

Introduction

When evaluating the results of a genome-wide association study (GWAS), it is important to perform a quality control to ensure that the results are valid, complete, correctly formatted, and, in case of meta-analysis, consistent with other studies in the same analysis. This package was developed to facilitate and streamline this process and provide the user with a comprehensive report. The process is divided into different phases, which are described in detail in this tutorial.

How to install GWASinspector

The easiest way to install the GWASinspector package is to get it from the Comprehensive R Archive Network (CRAN).

```
install.packages('GWASinspector')
```

In this method, the minimally required packages (see below) will also be downloaded and installed automatically.

You can also install `GWASinspector` using the source or binary package, which is available from our website (GWASinspector.com). In this case, you will have to install any required packages beforehand yourself.

```
install.packages('/path/to/package', repos = NULL, type = 'source')
```

Package dependency list

The following packages and modules are used inside `GWASinspector`. Some system modules are optional. However, it is suggested to install them to gain full functionality. You can check the availability of the optional items after installing GWASinspector by running `GWASinspector::system.check()` function.

Package name	Required	Description
ini	✓	Loads the configuration file
futile.logger	✓	Used for saving log messages
data.table	✓	Used for storing and manipulating input data
hash	✓	Used for translating header values
tools	✓	R base package
ggplot2	✓	Used for creating plots
knitr	✓	Used for writing reports in different formats
rmarkdown	✓	Used for writing reports in different formats
gridExtra	✓	Used for creating plot matrices
grid	✓	Used for creating plot matrices
RSQLite	✓	Used for storing reference files as a database
kableExtra	✓	Used for styling HTML report files
R.utils	✓	Used for reading gz files and getting file information
xlsx	✗	Used for creating excel report
pandoc	✗	See below
Java	✗	See below

Additional modules

- Pandoc is a free and open-source document converter that is used for creating an easy-to-view HTML report.

  Note: pandoc is automatically installed with RStudio.

  Installation: Please follow the instructions on how to install pandoc on your system. You can also use the 'installr' package on **Windows** operating systems. First, install the 'installr' package and then run the following command.

  ```
  install.packages('installr')

  installr::install.pandoc()
  ```

  On most Linux distributions you can simply run the below command for installation. Please note that the user should have root privileges:

  ```
  sudo apt-get install pandoc
  ```

- Java is required for `'xlsx'` package to work properly.

- xlsx package is used for generating the report file in Excel format. You can install it with the following command. Java Environment is required and should be installed first.

  ```
  install.packages('xlsx')
  ```

Auxiliary files

The header translation table

Correct recognition of columns is very crucial for any software and in most of the analytical tools users are asked to define the name of the columns if they differ from the standard names known to the software. Contrary to that, GWASinspector uses a translation table file for this purpose which handles column recognition. This table includes the most frequently used names in analytical applications and can easily be updated if needed. Another advantage of this approach is that files with different column names can be easily inspected together.

The header translation table is used to translate the dataset's column names (the header) into the standard names used by `GWASinspector`. A sample file is provided as part of the package, which can be modified according to your requirements. The file contains a two-column table, with the **left** column containing the standard column names used by `GWASinspector`, and the **right** column the alternatives ones from the GWAS results files. Both the standard and alternative columns must be fully **capitalized** and separated by a **tab** character.

You can obtain a sample file that includes most common variable/header names with the following command:

```
get.headerTranslation('/path/to/referenceFolder')
```

Note: Duplicated entries and empty lines in the header translation table are NOT accepted and will cause an error.

Example:

Correct file:

EFFECT_ALL	A1
EFFECT_ALL	ALLELE1
EFFECT_ALL	COD_ALL
EFFECT_ALL	CODED_ALL
EFFECT_ALL	CODED_ALLELE
EFFECT_ALL	EFFECT_ALLELE
EFFECT_ALL	EFF_ALL
OTHER_ALL	A2
OTHER_ALL	NONEFF_ALL
OTHER_ALL	ALLELE2
OTHER_ALL	NON_COD_ALL
OTHER_ALL	NON_CODED_ALL
OTHER_ALL	NON_EFFECT_ALLELE
…	

Wrong file because of a duplicated entry and an empty line

EFFECT_ALL	A1
EFFECT_ALL	ALLELE1
EFFECT_ALL	COD_ALL
EFFECT_ALL	CODED_ALL
EFFECT_ALL	CODED_ALLELE
EFFECT_ALL	EFFECT_ALLELE
EFFECT_ALL	EFF_ALL
OTHER_ALL	A2
OTHER_ALL	NONEFF_ALL
OTHER_ALL	ALLELE1
OTHER_ALL	NON_COD_ALL
OTHER_ALL	NON_CODED_ALL
…	

The standard allele reference file

The standard allele reference file is used to check the alleles in the datasets and to ensure that they are all in the same configuration (same strand, same coded alleles) in the post-QC data. This file can be in different formats, including .txt, .csv, .dat, .rds and .rdata files, and should contain the following columns:

**Column name**	**Description**
hID	The harmonized ID of a variant. The structure of this item is [Chromosome:Position:VT], where VT (variant type) can be either 1 for SNPs or 2 for INDELs.
ID	rsID of the variant (optional)
REF	Reference allele
ALT	Alternate allele
AF[#]	Allele frequency of the alternate allele

[#] Some reference panels have multiple population level AF values. In this case, the population string should be included in column name (EUR for European population, AFR for African population, etc.).

The following reference files are currently available from our website:

Reference dataset	Genome build	Number of variants
1000 Genomes Project [1]	GRCh37	84,346,970
dbSNP_GRCh37p13 [2]	GRCh37	82,798,854
dbSNP_GRCh38p7 [3]	GRCh38	82,285,539
HRC_r1-1 [4 *]	GRCh37	40,176,563
UK10K_COHORT_20160215 [5]	GRCh37	46,023,417
TOPMED [5]	GRCh37	162,739,927
HapMap_CEU_r28_b36 [6]	NCBI36	4,026,340

Source files available from:

[1] ftp://ftp.1000genomes.ebi.ac.uk/vol1/ftp/release/20130502/

[2] ftp://ftp.ncbi.nih.gov/snp/organisms/human_9606_b151_GRCh37p13/VCF

[3] ftp://ftp.ncbi.nih.gov/snp/organisms/human_9606_b151_GRCh38p7/VCF

[4] ftp://ngs.sanger.ac.uk/production/hrc/HRC.r1-1/

[5] ftp://ftp.ensembl.org/pub/data_files/homo_sapiens/GRCh37/variation_genotype/

[6] ftp://ftp.ncbi.nlm.nih.gov/hapmap/

* Contains rsID

The alternative allele reference file

The optional alternative allele reference serves as a back-up to the standard one. Variants that aren't found in the standard reference will be compared to the alternative reference. If they aren't in the alternative reference, they will be added to it. A check against an alternative reference is particularly useful before combining multiple GWAS results files in a meta-analysis. Mind that the order of the input files is important, because for the first occurrence of a variant that is not found in the standard reference, the data are considered the correct data for this variant (an assumption that might not be correct).

The alternative reference has six required columns: hID, REF, ALT, AF, SOURCE, and DATE_ADDED, for the harmonized variant ID (i.e. chromosome:position:type), reference allele, alternate allele, alternate allele frequency (AF), source and date added, respectively. In both reference files, the alleles must be aligned to the positive strand.

Note: The alternative reference data set may be updated by the QC function (unlike the standard reference data).

The effect-size reference file

An optional reference file is used for comparing the reported variants' effect sizes in the GWAS result file with those from earlier GWASs for the same phenotype. The correlation between the variants' effect sizes is calculated and a scatter plot is generated and saved in the output folder. Check here for more detail on the interpretation of the reported values.

Note: This file has five required columns: hID, EFF_ALL, NON_EFF_ALL, EFFECT, PVALUE

Note: A new effect-size reference file can be generated from an earlier GWAS result file (e.g. downloaded from GWAS catalog website) via the `run.inspector` function. The process is just like inspecting a result file, but with a modified setting in the configuration file.

1. Copy the file in the input data folder.
2. Set the filename, input and output paths accordingly in the configuration file.
3. Use the correct `standard allele reference` file for checking the result file. This should be the same reference file that will be later used for inspecting GWAS result files. (e.g. 1KG, dbSNP, …)
4. Add the below line in `[output_parameters]` section of the configuration file.

   ```
   save_as_effectSize_reference = TRUE
   ```

   Plotting and saving of the cleaned result files can be skipped if you are only interested in generating the effect-size reference dataset.

   ```
   save_final_dataset = FALSE
   make_plots = FALSE
   ```

5. Load the configuration file and run it through `run.inspector` function.

A new file will be generated in the output folder in **RDS** format (`*_effect-size_ref.rds`), which can be used as the effect-size reference dataset for future inspection of GWAS result files. This file can be renamed and then copied in the reference files folder (`dir_references`). Its full name (including the extension) should be set for the (`beta_ref_std`) parameter in the configuration file.

Available functions

Note: you can run `?GWASinspector in R` to get an overview of all functions in this package.

get.config()

Using this command, you can save a sample configuration file on your computer. Items in the file should be edited according to your needs and are described in the next section.

Arguments:

`dir.path` Directory to save the "config.ini" file.

Example:

```
get.config(dir.path = '/path/to/folder')
```

get.headerTranslation()

Using this command, you can save the default column-header translation table file. Items in the file should be edited according to your needs. Refer to this section for more information about this file.

Arguments:

`dir.path` Directory to save the "alt_headers.txt" file.

Example:

```
get.headerTranslation(dir.path = '/path/to/referenceFolder')
```

system.check()

Checks if required and optional packages are installed on the system. Although the optional packages do not contribute to the QC itself, having them available will allow for Excel and HTML formatted log files, which are easier to read and interpret.

Example:

```
system.check()
```

setup.inspector()

This function imports a QC-configuration file into R, and then generates an object of the `Inspector` class. This object canthen be passed to `run.inspector` function to perform the QC.

setup.inspector() requires a configuration (ini) file (see above). The function will also warn of any problems encountered when reading and validating this file.

Arguments:

`config.file`	Path to the configuration file on your computer.
`validate`	This option is only used for testing and debugging purposes and indicates whether to validate the configuration file. The default value is TRUE and it is strongly recommended to not turn it off for real jobs.

Example:

```
job <- setup.inspector(config.file = '/home/user/config.ini')

print(job)

## or simply

job
```

run.inspector()

This is the main function of the package, which runs the QC algorithm on GWAS result files. It requires an object of class "Inspector", which is created by [`setup.inspector`] function. Progress of the algorithm is printed to the R terminal and also saved in a log file. Printing of all messages inside the terminal can be suppressed by the `suppressMessages()` function without any effect on the log file.

Arguments:

`inspector`	An object of class "inspector", which is created by the `setup.inspector` command.

`verbose` If set to FALSE, no messages will show up in the terminal and are only saved in the log file.

`test.run` If set to TRUE, a test run is performed on the first 1000 lines of the input file. No plots will be generated.

Example:

```
job <- setup.inspector("/home/user/config.ini") ## create the object

job ## check the parameters

job <- run.inspector(job) ## run the algorithm
```

result.inspector()

This function displays a brief report about the results of running the inspector algorithm on a list of GWAS result files. The full report including plots, cleaned files and summary statistics are saved in the output folder during the algorithm run.

Arguments:

`inspector` An object of class "inspector" which has already been inspected.

Example:

```
job <- setup.inspector("/home/user/config.ini") ## create the object

job ## check the parameters

job <- run.inspector(job) ## run the algorithm

result.inspector(job) ## check the results
```

demo.inspector()

This function runs the algorithm on a fabricated GWAS result file. Users only need to set the path to an existing output folder, for saving the report files. The input file and reference dataset are embedded in the package.

Example:

```
demo.inspector('/home/user')
```

man.plot()

A function to generate Manhattan plots of a dataset.

Arguments:

Name	Type	Default Value	Description
`Data`	object		Data frame or data table containing the below columns
`Chr`	string		Name of chromosome column
`Pvalue`	string		Name of the p-value column
`Position`	string		Name of the position column
`Beta`	string	NULL	(optional) name of the effect-size column
`std.error`	string	NULL	(optional) name of the standard error column
`filename`	string		Full name and path of file to be saved (file extension should be 'png'). [e.g. "c:/users/researcher/study/man_plot.png"]
`plot.title`	string	'Manhattan Plot'	Title of the plot
`plot.subtitle`	string	''	Subtitle of the plot
`sig.threshold.log`	numeric	8	The -log10 transformed significance threshold, used for plotting a threshold line (e.g. 8 = 10^-8)
`p.threshold`	numeric	0.01	Threshold for plotting variants (i.e. p-values > 0.01 will not be plotted). Setting a higher threshold will significantly increase plotting time
`check.columns`	logical	TRUE	Whether to check input columns for invalid values

Examples:

```
Input.data <- fread("/path/to/resultFile")

man.plot(data = input.data,
      chr='Chromosome',
      pvalue= 'P_value',
      position = 'Position',
      fileName='/home/document/sampleFolder/m_plot.png')

man.plot(data = input.data,
      chr='Chromosome',
      pvalue= 'P_value',
      position = 'Position',
      fileName='/home/document/sampleFolder/m_plot.png',
      plot.title = 'Manhattan plot',
      plot.subtitle = 'sample research result',
      sig.threshold.log = 8,
      p.threshold = 0.01,
      beta= 'EFFECT',
      std.err= 'STD_ERR')
```

Output file naming conventions

In order to perform a comprehensive QC, GWASinspector, produce a series of tables and graphs. This section details which files are produced and what their purpose is.

Note: Keep in mind that only some of the following files may be generated and saved, depending on the configuration parameters and the integrity of input data.

Naming convention for a saved file is:

```
filename_output_tag +

name of input file without extension +

file_description +

extension
```

Notes:

- `filename_output_tag:` this is set in the configuration file (default value is QC)
- `file_description:` this describes the type of the file and its content. The following tables indicate possible values.

`Examples:`

- `results1_cohort1_graph_EAF_SR.png`
- `results1_cohort2_SNPs_improbable_values.txt`

Note: the main log and report files are always saved with the following names (overwriting any previous files with the same name).

- `[filename_output_tag]_log.txt`
- `[filename_output_tag]_Report.txt`

Main output, report and log files

File description	extension	description
log	txt	Summary of the QC process steps including any warnings
report	txt / HTML / xlsx	Report of the QC process
input_file_name	txt or txt.gz	Cleaned results file
object	rds	QC results as R data object

Lists of incorrect variants encountered during QC

File description	extension	description
vars_invalid_allele	txt	Variants with invalid alleles
vars_removed	txt	Variants with at least one missing or invalid value in a crucial column
vars_improbable_values	txt	Variants with at least one invalid value in a non-crucial column
vars_duplicates	txt	Variants with duplicated IDs
vars_monomorphic	txt	Monomorphic variants
vars_mismatches_BA	txt	Bi-allelic variants whose alleles did not match the reference
vars_mismatches_MA	txt	Multi-allelic variants whose alleles did not match the reference
vars_ambiguous	txt	Check “phase 3h” from this section

Graphs

File description	extension	description
graph_M	(png|jpeg|tiff)	Manhattan plot
graph_histogram	(png|jpeg|tiff)	Histogram of input data variables
graph_EAF_SR<br>graph_EAF_AR	(png|jpeg|tiff)	Scatter-plots of observed vs. allele frequencies in the standard/alternative allele reference
graph_p_correlation	(png|jpeg|tiff)	Scatterplot of expected vs. observed p-values
graph_QQ	(png|jpeg|tiff)	QQ plots
beta	(png|jpeg|tiff)	Scatter-plot of observed vs. reference effect-sizes

Checkgraph_effect-size	(png|jpeg|tiff)	The effect-size comparison plot
Checkgraph_precision	(png|jpeg|tiff)	The precision plot
Checkgraph_skew_kurt	(png|jpeg|tiff)	The skewness & kurtosis plot

Setting QC parameters in configuration file

GWASinspector uses a configuration (.ini) file to configure the QC algorithm. A sample file is included in the package, which can be used as a template (refer to `get.config` function tutorial). This file is edited by the user and then processed by the `setup.inspector` function. Please refer to the function's tutorial for more detail.

This file has three components:

1. Key or property: these are the basic parameters for configuring the QC. Every key has a name and a value, delimited by an equals sign (=).
2. Sections: Keys are grouped into named sections. The section name appears on a line by itself, in square brackets ([and])
3. Comments: Semicolons (**;**) or hash (**#**) at the beginning of the line indicate a comment. Comment lines are ignored by setup.inspector.

Notes:

- Key-names and section-names should **NOT** be edited or renamed. Otherwise the algorithm will not work properly.
- Lines that start with '**#**' character are comments about a parameter. Do **NOT** un-comment the information line by deleting this character.
- Properties that start with '**;**' character are commented parameters. In this case, the default value for that parameter is used. You can un-comment the parameter and set a value by removing the semicolon and changing the value after '=' sign.
- Avoid duplicated keys.

Configuration file sections and properties

Every part of the QC is configured in this file.

Section	parameter	default value	Description
paths	filename		the name of the file(s) to be QC'ed (see below for explanation).
	filename_output_tag	QC	output files will be prefixed with this tag.
	dir_data		the folder where input files are located.
	dir_output		folder where results are saved.
	dir_references		folder where reference and header files are located.
supplementaryFiles	header_translations		path to header file
	allele_ref_std		path to the standard allele reference file
	allele_ref_std_population		Which population data of the standard reference should be used. If the file has only one population, COMMON should be used.
	allele_ref_alt		path to alternative reference file
	beta_ref_std		path to Beta (Effect-size) reference file
input_parameters	column_separator	["\t" , "," , ";" , SPACE]	Characters that used as column separators in the input files
	na.string	["NA", "nan", "NaN", "."]	Characters used as missing values in the input files
	imputed_T	["1", "TRUE", "T", "YES", "Y"]	Character used to indicate TRUE/FALSE in Imputation Status column
	imputed_F	["0", "FALSE", "F", "NO", "N"]	
	effect_type	BETA	Beta or Odds Ratio? [BETA or OR]
output_parameters	save_final_dataset	TRUE	should the post-QC dataset be saved?
	gzip_final_dataset	FALSE	should the post-QC dataset be compressed?
	out_header*	standard	translation table for the column-names of the output file.
	out_sep	\t	character-strings of the column-separator
	out_na	NA	character-string to use for missing values
	out_dec	.	character string to use for decimal points

	html_report	TRUE	save final report in HTML format
	object_file	TRUE	save an R object containing QC results from each input result file
	add_column_multiallelic**	FALSE	Marking multi-allelic variants in the cleaned result file.
	add_column_AFmismatch***	FALSE	Marking variants with a high allele frequency difference in the cleaned result file.
	add_column_rsid⁺	FALSE	Adding rsID column to the output result files.
	add_column_HQ++	FALSE	Marking high-quality variants
	ordered	FALSE	Ordering the output cleaned files on CHR:POSITION value
	save_as_effectSize_reference	FALSE	Generates an effect-size reference dataset from the input file.
remove_chromosomes	remove_X	FALSE	whether X-chromosome, Y-chromosome, pseudo-autosomal and mitochondrial SNPs are removed
	remove_Y	FALSE	
	remove_XY	FALSE	
	remove_M	FALSE	
plot_specs	make_plots	TRUE	should the various QC steps create and save plots?
	graphic_device	png	file format for saving plot files (png, jpeg, tiff)
	plot_title		this title is displayed at the top of plots (only for single-file QC)
	plot_cutoff_p	0.01	Threshold for excluding low-significance SNPs from the QQ & Manhattan plots. Increasing this value will drastically slow QC.
filters	HQfilter_FRQ	0.01	Allele frequency threshold value for the high-quality (HQ) variant selection
	HQfilter_HWE	1e-6	HWE-pvalue threshold value for the high-quality (HQ) variant selection
	HQfilter_cal	0.95	Call rate threshold value for the high-quality (HQ) variant selection
	HQfilter_imp	0.3	Imputation quality threshold value for the

			high-quality (HQ) variant selection
	threshold_diffEAF	0.15	If the difference between reported allele frequency and the frequency in the reference file(s) exceeds this amount, the variants will be reported.
	minimal_impQ_value	-0.5	The minimal and maximal possible (i.e. non-invalid) imputation quality values.
	maximal_impQ_value	1.5	

* Check the below section for more information.

** As determined by comparison with the standard reference dataset. A new column named "MULTI_ALLELIC" is added to the cleaned result file, which contains either 0/1 or NA (if variant was not found in the standard reference).

*** The threshold_diffEAF parameter is used as the difference threshold. A new column named "highDiffEAF" is added to the cleaned result file which contains either 0/1 or NA (if variant was not found in the standard reference).

+ A new column "REF_RSID" will be added to the output result files. Make sure that the reference file has the rsID data.

++ A new column is added "HQ" marking high quality variants (either 0/1). Check phase3f from this page for more detail.

Renaming the column names:

Column names can be renamed automatically in the cleaned result files for compatibility with other analytical applications. Available standard formats are:

- "standard" (default setting) retains the column names used by inspect function
- "GWAMA", "PLINK", "GenABEL" and "META" set the column-names to those used by the respective program. Columns not used by those programs retain the standard names.

GWASinspector	GenABEL	GWAMA	PLINK	META
MARKER	name	MARKER	SNP	rsid
CHR	chromosome	CHR	CHR	chr
POSITION	position	POSITION	BP	pos
STRAND	strand	STRAND	STRAND	strand
EFFECT_ALL	allele1	EA	A1	allele_B
OTHER_ALL	allele2	NEA	A2	allele_A
EFF_ALL_FREQ	effallelefreq	EAF	EFF_ALL_FREQ	EFF_ALL_FREQ
N_TOTAL	n	N	N	N
EFFECT	beta	BETA	BETA	beta
STDERR	sebeta	SE	SE	se
PVALUE	p	P	P	P_value
CALLRATE	call	N	N	N
HWE_PVAL	pexhwe			

Selecting input names using the filename key

Input files should be in the `'dir_data'` folder and are selected using the `'filename'` parameter in the configuration file. This parameter serves as a template and can be used to load multiple files. This command is case-insensitive (e.g. File or FILE or file are identical).

Regular expression can be applied in this parameter to filter or select the input files. The following table provides some sample filters. You can refer to standard RegEx tutorials for more complex patterns.

Example:

command	Description	Example of successful matches
filename = txt	All files in the dir_data folder that have 'txt' in their name will be selected.	gwas_file.txt<br>gwas_file.txt.gz<br>samples_txt_file.csv
filename = txt$	All filenames that end with 'txt' will be selected.	gwas_file.txt
filename = gz$	All filenames that end with 'gz' will be selected.	gwas_file.txt.gz
filename = ^gwas.+txt$	All filenames that start with 'gwas' and end with 'txt' will be selected.	gwas_file_h9v11.txt<br>GWAS_sample_file.txt
filename = ^gwas.+txt.gz$	All filenames that start with 'gwas' and end with 'txt.gz' will be selected.	gwas_sample1.txt.gz<br>gwas_sample2_file.txt.gz
filename = ^(gwas|ewas).+txt$	All filenames that start with either 'gwas' or 'ewas' and end with 'txt' will be selected.	ewas_file_No2.txt<br>gwas_sample_file.txt
Filename = ^(gwas|ewas).+(txt|dat)$	All filenames that start with either 'gwas' or 'ewas' and end with either 'txt' or 'dat' will be selected.	ewas_file_No2.txt<br>gwas_sample_file.txt<br>ewas_study__h1v11.dat
filename = ^(gwas|ewas).+(v10).+txt$	All filenames that start with either 'gwas' or 'ewas', have 'v10' in the middle and end with 'txt' will be selected.	ewas_file_V10.txt<br>gwas_v10_Cleanedfile.txt

Step-by-step guide to run a QC

System requirements:

This package requires R (> 3.2) and is tested on popular operating systems (e.g. Microsoft Windows, Ubuntu, CentOS, macOS). System requirements depend on the size of the result files and references. A PC with Intel Core i5 CPU or equivalent (> 2.4GHz) and at least 36 GB of RAM is recommended for analyzing a set of routine GWAS result file. A 64-bit operating system is not required but strongly recommended.

Prepare:

1- Input folder: for storing the input result files
2- Output folder: for saving the QC output files and reports
3- Reference folder: for keeping the reference datasets and header-translation table
    - Allele frequency reference datasets can be obtained from our website.
    - A sample header-translation table is embedded in the package. Refer to `get.headerTranslation` function for more detail.
4- Configuration file: for setting the run-time parameters and above-mentioned folder paths. A sample file is embedded in the package that should be used as a template. Refer to `get.config` functions and section "Setting QC parameters in configuration file" for details.

Step 1: Load the package

After installation, try loading the package with the following command.

```
library(GWASinspector)
```

Step 2: Check R environment

Run the following function to check if required and optional libraries are available. Refer to the package dependency list (above) for detail about mandatory and optional libraries.

```
system.check()
```

Step 3: Download the standard allele frequency reference datasets

Standard allele frequency reference datasets are available from our website. This file should be unzipped and copied in the references folder [`dir_references`] property of the configuration file.

Step 4: Get the header-translation table

A sample header-translation table can be obtained with the get.headerTranslation() function. Refer to function manual and this section for more information about this file.

```
get.headerTranslation("/path/to/referenceFolder")
```

Step 5: Get the configuration file

A sample configuration file can be obtained with the get.config() function. Refer to function manual for information.

```
get.config("/path/to/folder")
```

Step 6: Modify the parameters in the configuration file

Please refer to the previous section for full detail on how to modify the parameters and default values. Parameters in this file are used for reading input files, analyzing the data and saving the reports. There are multiple lines of comment and information about each parameter (lines that start with # and ; are comments and sample possible parameters, respectively). You should only change the lines that contain a key according to your specific needs.

Step 7: Run the QC function

The QC is configured by the ini file, which is imported into R through `setup.inspector` and turned into an object of the Inspector class. To perform the QC, process the object with `run.inspector`. A quick scan of the results can be performed via `result.inspector`, but the primary outcome of the QC are the log files and graphs generated by `run.inspector`. An exhaustive log file indicating the progress and possible warnings is also saved which can be used for localization of any problems during this run.

```
job <- setup.inspector("/home/user/config.ini") ## create the object
job ## check the parameters
job <- run.inspector(job) ## run the algorithm
```

Note: You can also perform a test-run on the first 1000 lines of result files. Please refer to the section on `run.inspector` function for more detail.

Quality control steps of GWAS result files

The algorithm will process all selected files, one by one, through the following stages.

1. Loading
    a. Loading the dataset
    b. Translating the column headers to the standard names
2. Data integrity & SNP check
    a. Removing variants with missing/invalid crucial values
    b. Removing duplicated variants
    c. Removing monomorphic variants
    d. Removing allosomal or mitochondrial variants
    e. Checking missing/invalid values for non-crucial variables ("improbable variants")
    f. Generating a harmonized ID for each variant
3. Aligning alleles with reference and comparing the allele frequencies
    a. Negative strand switch
    b. Comparing input file with a reference dataset
    c. Flipping of alleles
    d. Switching of alleles
    e. Tagging palindromic variants
    f. Tagging High-Quality (HQ) variants
    g. Removing mismatched variants
4. Quality control of the other statistics
    a. Genomic control lambda, Visscher's statistic, skewness, kurtosis calculation
    b. Checking P-value correlation between expected and observed values
    c. Calculating allele frequency correlation
    d. Creating histograms, scatterplots, QQ plots and Manhattan plot
5. Creating the quality control summary statistics report as HTML, excel and text files for easy interpretation of the results
6. Saving the cleaned dataset
7. Comparing multiple GWAS results files (if the algorithm is run on a series of files)

Phase 1: Loading the dataset

The QC process starts with reading the GWAS result input file(s).

Phase 1a: finding input files

Input files should be put in the `'dir_data'` folder and they are selected using the `'filename'` parameter in the configuration file. Refer to this section for details.

***Phase 1b**: importing the data*

All tabular text files can be selected and loaded. This includes '.txt' , '.dat' and '.csv' files in either text or compressed ('zip' or 'gz') format. Loading zipped files takes more time than loading the unzipped version of the same file. In general, gzip files (e.g. *.txt.gz , *.csv.gz) are recommended over zip files format (*.zip), due to:

- faster loading
- by default, the name of the file inside the zipped package (.zip file) and the zipped file should match (for example, **sample_file_no2.zip** should contain **sample_file_no2.txt** file). This is not an issue for gzip files.

Input files should contain a header row and all columns should be separated using an identical column separator character. Tab character (`\t`) is selected by default. If your input file uses another character as column delimiter, this should be defined by setting the `column_separator` parameter in the configuration file.

***Phase 1c:** checking column headers*

The algorithm requires the dataset to have all standard crucial columns for running the QC. Translating the data column names into below standard names is done using the header translation table (check here). The path to this table is defined in the `header_translations` argument of the configuration file.

Note: Input file will be excluded from further analysis if there are duplicated column names or any of the crucial columns are absent. Columns whose name cannot be translated ("unknown columns") are ignored from further analysis, but will be preserved in the output file.

Table 1: columns that are analyzed during QC process, their valid values, and whether they are crucial

Column name	Description	Valid value	Crucial
CHR	Chromosome	1-26 , x , y , xy , mt , X, Y, XY, MT	✓
POSITION	Variant position on chromosome	Integer numbers	✓
EFFECT_ALL	Effect or Reference allele	G,C,T,A,I,D,-,0,R	✓
OTHER_ALL	Other or Alternate allele	G,C,T,A,I,D,-,0,R	✓
BETA | OR	Effect size (either beta or odds ratio)	Numeric value	✓
STDERR	Standard error	Numeric value	✓
MARKER	Variant marker (e.g. rsID, hID)	string	×
STRAND	Which strand	+ or -	×
PVALUE	P-value	Numeric value	×
EFF_ALL_FREQ	Allele frequency	Numeric value	×
HWE_PVAL	HWE P-value	Numeric value	×
IMP_QUALITY	Imputation quality	Numeric value	×
IMPUTED	Variant is imputed or not	List of numbers (default: 0-1). Can be set in the configuration file	×
CALLRATE	Call rate	Numeric value	×
N_TOTAL	Number of samples	Numeric value	×

Phase 2: Data integrity & SNP check

The aim of this step is to check that all variants have the required data and that all columns only contain valid values. Count of all removed lines from the following steps are save in the log and report files.

This phase is divided into below sections:

Phase 2a: *Removing variants with missing/invalid crucial values*

Variants with an invalid or missing value in any of the crucial columns are removed from the dataset and the first 100 variants are saved as a text file in output folder (check the "Report files" section of this tutorial for further information).

Phase 2b: *Removing duplicated variants*

Variants with duplicated IDs and alleles are removed from the dataset. By default, the combination of chromosome, position, effect-allele and other-allele of the variants are checked for duplicated information. The first 100 duplicated variants are saved as a text file in output folder.

Phase 2c: *Removing monomorphic variants*

Variants with allele frequency of 0 or 1 and variants with identical alleles are monomorphic and will be removed from dataset.

Phase 2d: *removing allosomal and mitochondrial variants*

This step is optional and will delete variants on a specific chromosome (X or 23, Y or 24, XY or 25 , M/MT or 26). The default setting is to keep all variants; to change this, set the following parameters in the configuration file to TRUE:

- `remove_X`
- `remove_Y`
- `remove_XY`
- `remove_MT`

Phase 2e: *Checking missing/invalid values for non-crucial variables*

Variants with missing or invalid non-crucial values will be counted and reported.

Phase 2f: *Adding harmonized ID for each variant*

A harmonized ID will be generated for each variant from other variables. The structure of the hID is:

```
hID = Chromosome:Position:VT
```

🖉 Variant Type (VT) is either 1 for SNPs or 2 for non-SNP variants. This is determined by evaluating both alleles of a variant.

🖉 GWASinspector does not change the data in GWAS result files.
In case of missing or invalid values:

1- Variants with invalid or missing values in any of the crucial columns are removed from further analysis and will not be included in the final cleaned result file.
2- Variants with invalid or missing values in the non-crucial columns will be counted and reported to the user. But, no changes will be applied and they remain intact.

Two sample files including variants with missing values in the crucial and non-crucial columns are saved separately in the output folder for further investigation. Only the first 100 variants are saved, as this would be sufficient to pinpoint the possible problems in the result file and to prevent high disk usage.

Phase 3: Aligning alleles with reference and comparing the allele frequencies

The aim of this step is to make sure that the reported allele frequency is for the correct allele (effect allele) and of the expected order. This is accomplished by matching the data with a reference panel (e.g. HapMap, 1000G) and then adjusting the alleles. Allele matching for indel and multi-allelic variants are also done in GWASinspector. Available variant alleles and their frequency are stored in the supplied reference files for this purpose (below table). Each record in the table has the information for one variant. Records for multi-allelic variants were generated from multiple lines of data in the original study files (if not already done).

CHR	POS	REF	ALT	AF
1	774785	G	A,C	0.994,0.001
1	751499	A	AAAT,AAT	0.0009984,0.01637

Note: By definition, a SNP is a change of a single nucleotide in the DNA sequence, whereas an indel incorporates or removes one or more nucleotides. Both types of variants could play an important role in complex traits and are of great importance in GWAS analysis.

Note: A SNP is called "bi-allelic" if it only has two alleles in the reference data. If more than two alleles occur, the SNP is described as "multi-allelic". The process of flipping and switching of the alleles is more complicated for multi-allelic variants. In this case, input variant alleles will be checked against the combination of alleles for the matched multi-allelic variant and then modified accordingly. A multi-allelic variant is considered a mismatch if none of the combinations are valid compared to the reference dataset. It is important to note that this process is dependent on the selected allele reference and there might be minor changes in different datasets (also check this page, phase 3g).

For example, in the below table:

- variant number 1 is a multi-allelic SNP that can be matched with the reference dataset.
- variant number 2 is a mismatched multi-allelic SNP.
- variant number 3 is a matched bi-allelic SNP.
- variant number 4 is a matched indel variant.
- variant number 5 is a mismatched indel variant.

GWAS result file

No.	ID	A1	A2	AF	BETA
1*	2:281886	T	G	0.42	0.284
2**	2:281886	T	A	0.34	0.166
3	1:16949	A	C	0.02	-0.12
4	8:436927:2	A	ATGG	0.31	-0.018
5	8:436927:2	A	AGG	0.23	0.076

Reference panel file

ID	REF	ALT	AF
2:281886	T	C,G	0.3767,0.334
1:16949	A	C	0.015
8:436927:2	A	ATGG	0.2873

* Green indicates matched variants

** Red indicates mismatched/ambiguous variants.

Phase 3a: *Negative strand switch*

The STRAND column is checked at first (if present) and negative-stranded variants will be "strand-switched". This means that an "A" allele becomes "T", "T" becomes "A", etc.

Phase 3b: *comparing input file with reference dataset*

Each variant is looked up in the reference dataset by the harmonized ID.

Phase 3c: *Flipping of the alleles*

Variants whose effect allele matches the reference major allele and non-effect allele matches the reference minor allele will be "flipped": their alleles are reversed, and their effect-size and allele frequency inverted (i.e. effect-size = 1.32, AF = 0.76 becomes effect-size = -1.32, AF = 1 - 0.76 = 0.24)

Phase 3d: *Switching of the alleles*

If a variant is found in the reference data but alleles do not match, function will attempt to fix this by carrying out a strand-switch. This assumes that variants are on the negative-strand, but have not been listed as such. These variants are counted and reported as switched variants in the output report.

🖉 Note: the process of flipping and switching of the alleles is more complicated for multi-allelic variants. Input variant alleles will be checked against the combination of alleles for the matched multi-allelic variant and then modified accordingly.

Phase 3e: *Tagging palindromic variants*

Palindromic variants are variants with a strand-independent allele configuration (i.e. A/T or C/G), and will be reported in the output report file.

Phase 3f: *Tagging High-Quality (HQ) variants*

High-quality (HQ) variants are selected through the settings specified by thresholds on allele frequency, call rate, HWE p-value and imputation quality values that are set in the configuration file.

🖉 Notes:

- The HQ filter does not *remove* SNPs from the dataset; it merely *excludes* them from a number of tests (including the Manhattan plot).
- The allele frequency filter is two-sided (check the below example).
- A variant is considered high-quality if it meets **all** quality-criteria
- The thresholds are inclusive; i.e. variants that have a value equal to or higher than the threshold will be included.

Example: by setting the below values in the configuration file, variant whose allele frequency is (above 0.01 **OR** below 0.99) **AND** HWE P-value is above 10^-6 **AND** call-rate is above 0.95 **AND** Imputation quality is above 0.3 will be considered as HQ variant.

- `HQfilter_FRQ = 0.01`
- `HQfilter_HWE = 1e-6`
- `HQfilter_cal = 0.95`
- `HQfilter_imp = 0.3`

Phase 3g: *removing mismatched variants*

Variants that are found in the reference dataset by hID matching but their alleles could not be matched after flipping or switching of the alleles are removed from dataset. The first 100 variants are saved as txt file in the output folder.

Note: Mismatched multi-allelic variants will be saved as a separate file for user observation.

Phase 3h: *removing ambiguous variants*

Variants that cannot be correctly matched due to the following conditions are removed from the result file (this file almost always includes non-SNP variants). The first 100 variants are saved as txt file in the output folder. Check here for example:

- INS and DEL on the same position with reverse alleles. This mimics a switched variant.
- Absence of alleles for a multi-allelic non-SNP variants (e.g. I,R,0,D).

Phase 4: Quality control of the other statistics

At this point no further changes will be made inside the file. The function first determines if there are sufficient non-missing, non-invalid values for the various QC tests.

Phase 4a: *Visscher's statistic, skewness and kurtosis calculation*

- Kurtosis is a measure of how well a distribution matches a Gaussian distribution. A Gaussian distribution has a kurtosis of 0. Negative kurtosis indicates a flatter distribution curve, while positive kurtosis indicates a sharper, thinner curve.
- Skewness is a measure of distribution asymmetry. A symmetrical distribution has a skewness of 0. A positive skewness indicates a long tail towards higher values, while a negative skewness indicates a long tail towards lower values. Ideally, one expects both the skewness and kurtosis of effect sizes to be close to 0. But in practice, these statistics can be hugely variable.
- Visscher's statistic is calculated by the following formula.

$$Median\left(\frac{1}{2 * FRQ * (1 - FRQ) * (SE)^2}\right) \Big/ N$$

FRQ = allele frequency, SE = standard error, N = sample size

All above values are separately calculated and reported for HQ/LQ variants.

Phase 4b: *Checking P-value correlation between expected and observed values*

Expected p-value for all variants are calculated from chi^2 = $\left(\frac{Effect}{Stderr}\right)^2$, which follows a chi-square distribution with 1 degree of freedom. This value is compared with the observed data in the column and the correlation between the two should be close to 1. If this isn't the case, either a column was misidentified when loading the data or the wrong values were used when generating the dataset.

Phase 4c: *Calculating allele frequency correlation*

The correlation between allele frequency of variants in the input file and the reference dataset are calculated for palindromic and non-palindromic variants. The correlation efficient should be above 0.9.

Phase 4d: *Creating histograms, scatterplots, QQ plots and Manhattan plot*

Phase 5: Generating the report

A separate report file will be generated for each input file. Also, depending on your computer's available modules and installed packages, HTML and Excel format of the report will be generated and saved in the output folder for easy interpretation of the results.

For the best user experience GWASinspector generates separate QC reports for each input result file in text, HTML and excel formats.

- Textual data are presented in structured tabular format with clear content and descriptions compared to the usual CSV files.
- Tabular data are saved in a multi-sheet Excel file. QC settings, individual file reports and between-study comparison reports are separately saved in identified sheets.
- As the best solution for sharing or examining all QC metrics in one place, standalone HTML files are provided to illustrate the textual and graphic reports. In addition to a separate HTML report for each result file, between-study comparison report is also saved in HTML format with hyperlinks which makes it very easy to navigate between the files.

Phase 6: Saving the cleaned dataset

The cleaned dataset will be saved in the output folder according to parameters in the configuration file. Check the detail on '`output_parameters`' section of the configuration file.

Phase 7: Comparing multiple GWAS results files

Important summary statistic and key metrics of input study files are compared together and saved in tabular format plus three graphs. This report help find significant anomalies or differences between the files.

1. *Precision plot*: As sample size increases, the standard error is expected to decrease. Hence, the precision (1 / median standard-error) is plotted against the square root of the sample-size for many studies, one a linear relation is expected.
2. *Skewness-kurtosis plot*: Generates a skewness vs. kurtosis plot for input result files.
3. *Effect-sizes box plot*: compares the distribution of effect-sizes between studies, sorted on sample size.

Interpreting the QC reports

The overall report

The first thing to look at when running GWASinspector is the output in the R console. This output will also be copied into a **log file** (`results1_log.txt`, in our example). There's a great deal of text in there, but most of it are status flags indicating the process of the QC. The only thing that you need to check is whether all the intended GWAS files were processed, and whether it flagged any WARNINGS or ERRORS.

After skimming through the log file, you should check the **QC report**. This is the main summary of the QC. Depending on your settings and system, the report will be saved in multiple formats (.txt, Excel and HTML). In our example, this would be `results1_report.txt, results1_report.xlsx`, etc. The report consists of two tables and, if multiple files were processed, three figures (incorporated in the HTML file, but also saved with the "`Checkgraph_`" prefix). The topmost table lists the processed files, and serves as a legendum for the below table and figures. In the HTML report, the table is also hyperlinked to the reports of individual files; in the Excel file, the individual file reports are on separate tabs. Regardless of the format chosen, GWASinspector will always produce individual .txt reports for each input file - the information in there is identical to that in the HTML and Excel reports.

The **multi-file report** is the second table of the QC report (and the third tab of the Excel file) and gives a side-by-side comparison of the QC results for all files. This information is a condensed version of the data given in the individual file reports (so for see below for how to interpret these) - the purpose of the table is to make it easier to spot substantial differences between the individual results files.

This is also the purpose of the three graphs. The topmost plot of the HTML file (`checkgraph_precision`) is a **precision plot**. Precision is the inverse of the median standard error (i.e. the uncertainty of the estimates): it's plotted against the square root of the sample size. As sample size increases, you would expect precision to increase proportionally. As such, you expect all your results to be on a roughly diagonal line.

🖉Note: This plot will not be generated if sample size is missing in the result file.

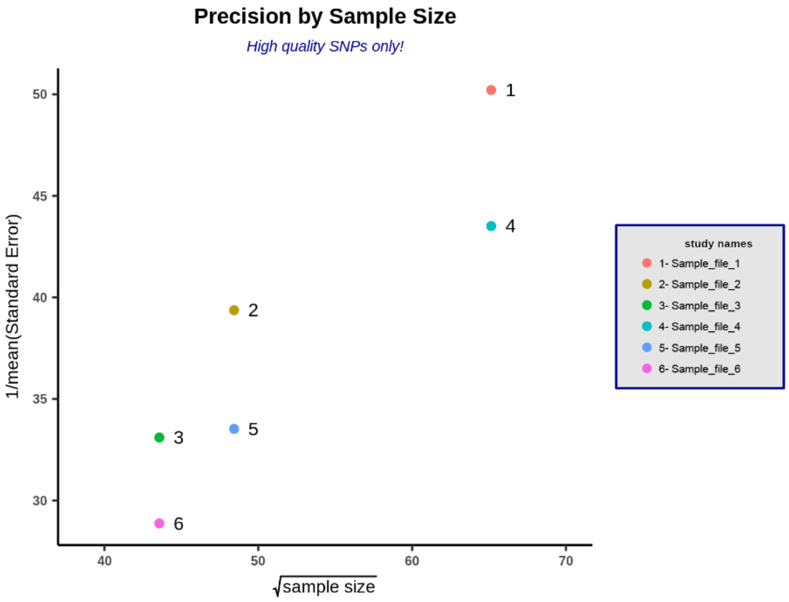

Figure 1: Sample precision plot. Dots are nicely located close to the diagonal.

In the below example (Figure2), however, there is one file that has a clearly higher precision than the others. It indicates that the file is not comparable to others, which means they cannot be combined in a regular meta-analysis. There are several possible causes for differences in precision: it could be that they analyses a different phenotype (or the same phenotype in a different unit or transformation), or a different model. In this case, the culprit was a different software program used in the analysis, which performed an undocumented standardization of the outcome values.

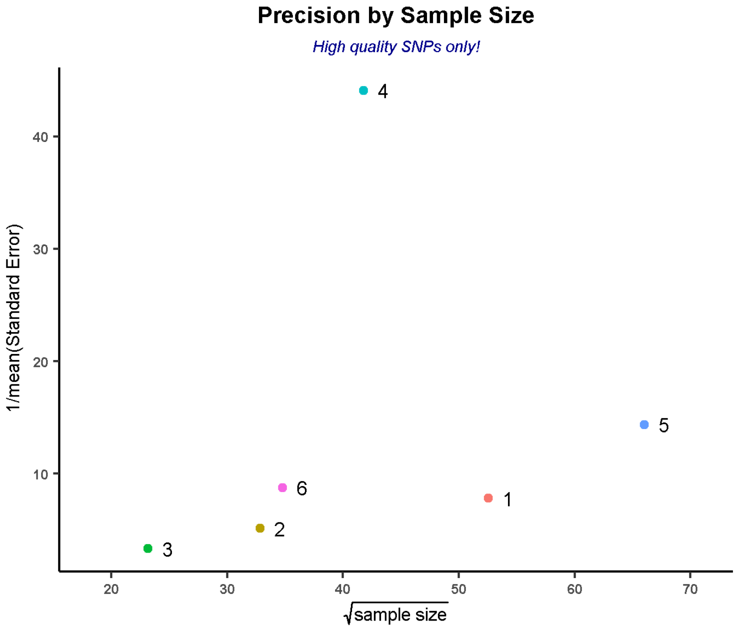

Figure 2: Precision plot that indicates a discrepancy in the file series for study 4.

The second, `Checkgraph_skew_kurt`, is a **skewness vs. kurtosis plot**. Skewness is a measure of distribution asymmetry; while kurtosis is a measure of how well a distribution matches a Gaussian distribution. Skewness values should be close to 0 and kurtosis values should be smaller than 10. If there are obvious outliers, there is something wrong with the effect-size distribution of that file, which will usually cause further issues that can be spotted in the QC report of that particular file.

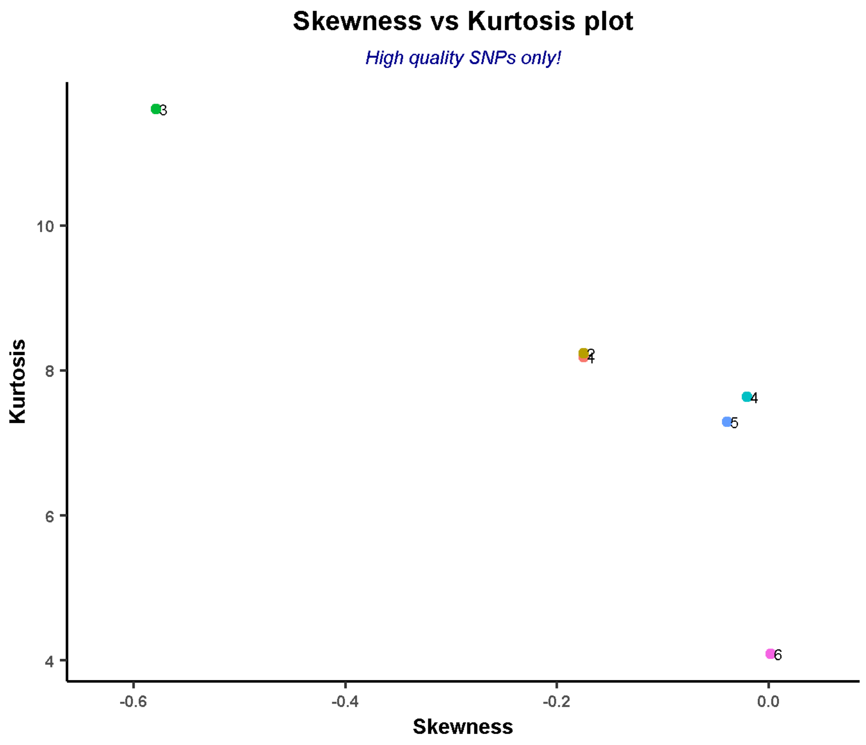

Figure 3: Sample skewness vs kurtosis plot. For study number 3 in the upper left corner of the graph both skewness and kurtosis deviate from the expectation and hence the effect sizes of this study should be checked.

The final plot, `Checkgraph_effect-size`, is a box-plot of effect-sizes. It compares the **effect-size range** (of high-quality markers only) of each input file, and sorts them by sample size. This serves the same purpose as the precision plot: with increased sample size you expect a reduced effect-size range because precision improves. If a file has notably wider or narrower range than expected, this strongly suggests that its results are not comparable to the others.

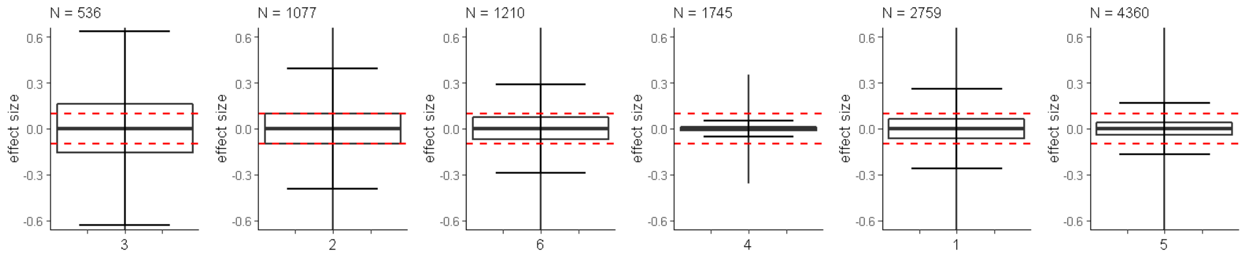

We see that file number 4 has a substantially narrower range than the other files. It's only the 3rd largest sample, yet it's by far the most precise. Again, this means that there is an issue with the effect-size distribution within this study.

The individual file report

The individual file report, and the accompanying plots, are the main product of the QC. The top of the report consists of a recap of the analysis settings, which is just there as a reminder. In the HTML file, this is followed by a table giving the translation of the column headers. Check if it reports a missing column (if it reports a missing column that should actually be present in the dataset, it was probably misspelled in the header translation table), and move on to the next table. In the Excel file, these will be on separate tabs of the overall report.

The next table, the **column report**, is a test of data integrity. It checks if the columns contain the values you would expect (standard errors above zero, p-values between 0 and 1, etc.). It's pretty common to see a small number of anomalies (standard errors rounded down to 0, and such) per column; and sometimes large numbers (up to 2/3 of the dataset) of effect-sizes and standard errors may be missing (i.e. the software did not produce results for this particular marker, so it simply gave a NA as result). Such outcomes are not problematic. What is problematic is if there are large numbers of *invalid* values; or entire columns are missing when they should be present. This indicates something went wrong either when formatting the dataset, or when GWASinspector translated the column names (see the table above).

Below that is the **variant processing** table. This table gives an overview of the number of variants in the dataset; and whether they are excluded during the QC process. Step 1 is the removal of duplicated variants and variants lacking an effect size (or odds ratio), standard error and/or p-value (or, alternatively, have an invalid value, such as a standard error of -1). As these values are the primary result of a GWAS, markers without these are essentially useless and can be safely removed.

The second step involves removing monomorphic markers (i.e. markers with an allele frequency of 0 or the same minor and major allele), as these results are uninterpretable. Allosomal and mitochrondial markers are also removed here if the configuration file specified this.

The third step is performed when the results file is matched to an allele reference. In order to interpret the effect sizes, it is necessary to know the effect (and alternative) allele. Therefore, GWASinspector will compare the results to an allele reference and check if the alleles match.

Following is a series of tables in the description of variants and the result from matching variants with reference datasets section - an overview of the remaining variants in the dataset. These are of little importance, though do check whether the majority of markers was matched with the allele reference. If a substantial number of variants did not appear in the reference - it suggests the wrong reference was used, or that your data uses a different marker naming convention.

The main outcome of note here are the *allele frequency correlation* r values. These indicate how well the reported allele frequencies match that of the reference. Ideally, you want this to be over 0.9. However, in small analysis samples, or when your population doesn't match the reference, the correlation can be much lower. That's not necessarily a problem, but negative values are. It indicates that the allele frequency is reported for the wrong allele. This in turn may mean that the effect and reference alleles have been switched; and that your effect-size is reported for the wrong allele as well. Scatterplots of reported and reference frequencies are also included to give a better view in how the frequencies are distributed (in our example, these would be named `QC_results_graph_EAF_SR.png`).

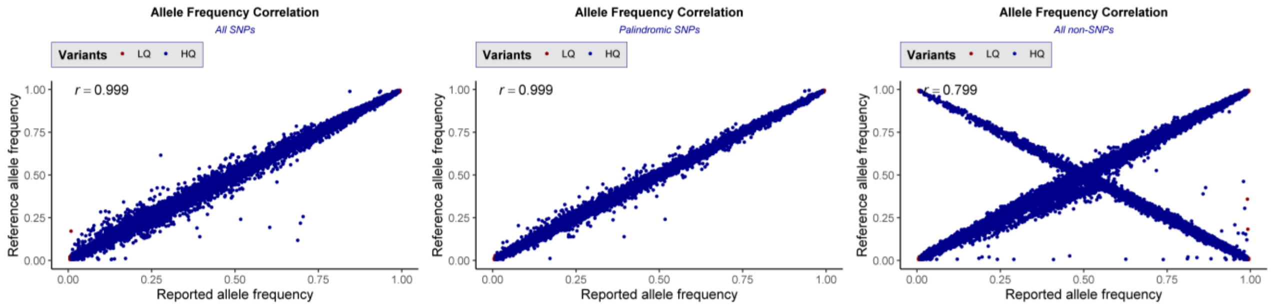

The scatter plot is split into 3 sections: SNPs, palindromic SNPs, and non-SNPs. The SNP correlations are fine, but a minority of non-SNPs appears to have the opposite allele frequency to the reference. This is troubling - if their frequency is the exact opposite of what is expected, either the wrong frequency is reported or the effect and non-effect alleles have been flipped (in the latter case the reported effect-size should be suspect as well). Also note that, while this problem did affect the allele frequency correlation, it is still fairly high. Merely looking at the tables wouldn't have revealed this - you need to check the graphs as well.

Of interest are also the palindromic SNPs: these are SNPs with the same allele on the positive and negative strand (i.e. A/T or C/G SNPs). Because of this, it is not possible to recognize whether palindromic SNPs are on the negative or positive strand. As such, if the correlation is much lower for these SNPs than for non-palindromic ones, there may be unrecognized strand-switching going on. Fortunately, that does not appear be the case here.

The final section of the file reports the **QC Summary statistics**.

The *p-value correlation* is a test of how well the reported p-value actually matches the effect-sizes and standard errors. If this is less than 1, or if the graph does not show an exact 1-to-1 scale; the p-values you have received are modified somehow. In case of the scale not being 1 to 1; this may be due to a genomic control correction. If not, the p-value doesn't belong to the effect size. Again, don't just look at the tables but check the plot as well:

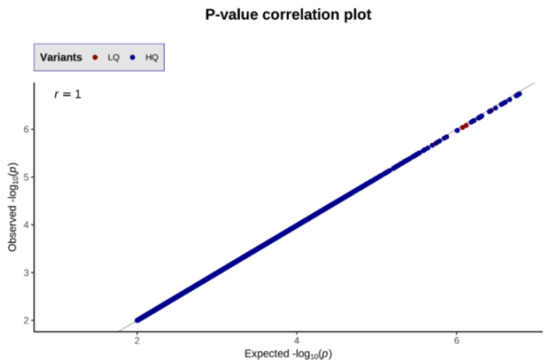

Figure 4: Sample P-value correlation scatter plot

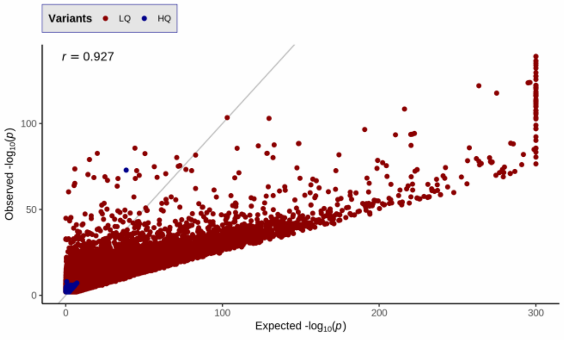

Figure 5: Sample of a possible problematic P-value correlation scatter plot

In the above example (Figure 5), the problem is related to the analysis software. Certain analysis models in SNPtest, for example, will show this problem for a subset of low-quality markers. We cannot explain this, but since the markers in question are low quality it's a minor problem anyway.

The next table covers the *data distribution* itself. The *lambda value* is also very important. It indicates p-value inflation; that is: how much more significant your p-value distribution is compared to a random p-value distribution. Ideally, this value should be 1. Values up to 1.1 are acceptable, but anything higher than that suggest some sort of population stratification. See also the QQ plots, below.

The final cells of this indicate whether HWE p-values, imputation quality, callrate and sample size are fixed. This won't affect the QC, but it may indicate laziness during formatting of the file. For example, sample size should be affected by callrate/imputation quality/genotyping uncertainty. If it is fixed, it suggests that the analyst simply assigned the max sample size to all variants without taking this into account.

The second distribution statistics table indicates the descriptive statistics for effect-size, SE, allele frequency, imputation quality, P-value and HWE P-value variables (if included in the result file). This table is divided in two: 1) statistics from including all variants 2) statistics by only including the HQ variants. Histograms of variables distribution are also generated to reveal any defect in the data. For example, histograms in figure 6 illustrate the anticipated distribution. Call-rate has been missing in this result file.

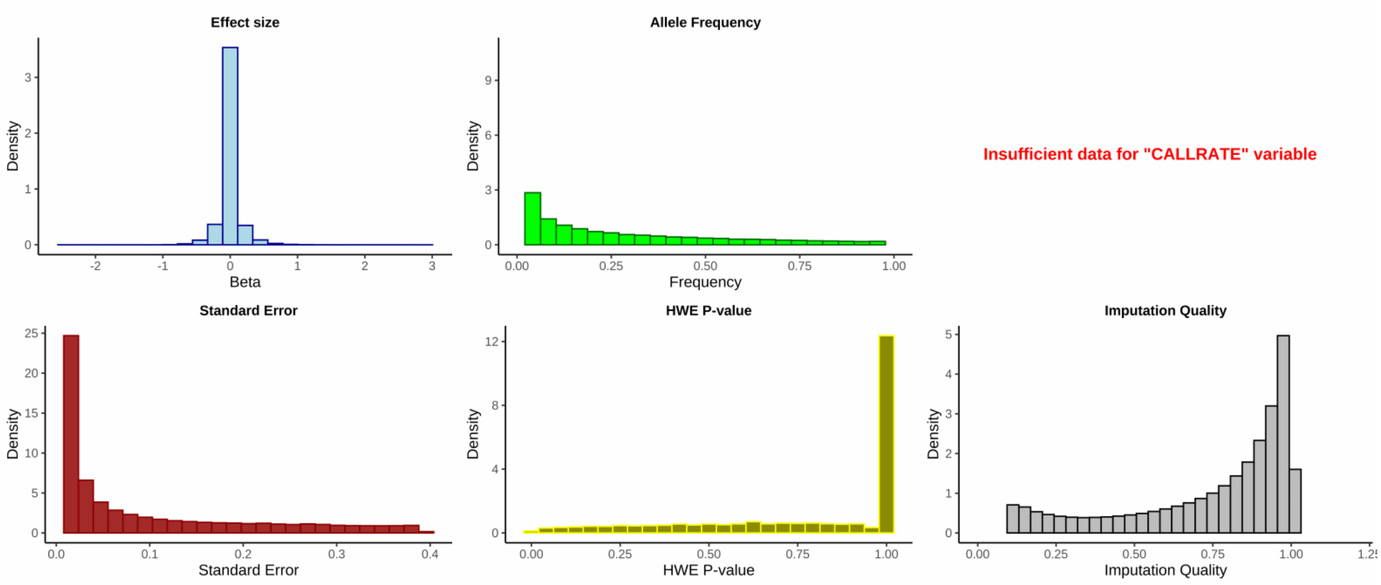

Figure 6: Histogram plots displaying variables distribution.

If an effect-size reference was provided, the effect-size correlation will be reported in the next section. This is used to check the concordance between the reported effect sizes coming from different studies.

Note: It is recommended to generate an effect-size reference dataset from previously published data (e.g. GWAS catalog), or if not available, from the study in the meta-analysis that is the most likely to be reliable. Refer to this section on how to generate the dataset.

The effect-size correlation test is a multi-step process, and in each stage a more rigorous P-value filter is applied (P-value < 10^{-3}, 10^{-4}, 10^{-5}, 10^{-6}). This filtering is applied separately on the reference dataset and on the result data. In total, eight correlation values are calculated and reported (4 filtering steps over 2 datasets). Only one scatterplot is generated to save time, which illustrates the data after filtering the variants with P-value < 10^{-4} in the reference dataset.

Based on the reported correlation values:

1- A high, positive value is expected, and suggests that the reported and reference datasets are harmonized (figure 7.1). This correlation is also expected to increase as more stringent P-value filters are applied.

2- A negative value indicates that the reported effect-sizes are for the wrong allele compared to the reference dataset (figure 7.2). In this case, one expects the more stringent filters to give stronger negative outcomes.

3- A low correlation value indicates a problem in the results file possibly due to a misidentified column name or a different allele reference. Alternatively, the analyses are not comparable (figure 7.3). There will not be any significant changes in correlation after more stringent filtering of the variants in the reference or result files.

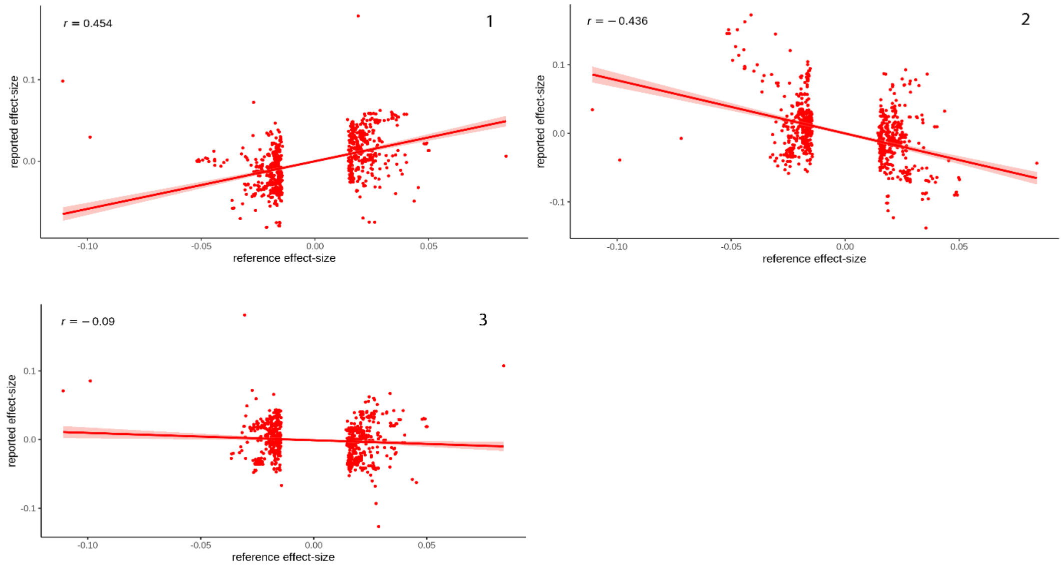

Figure 7. Sample effect-size correlation scatter plots

At the bottom of the report are the QQ plots and Manhattan plots. The QQ plots (figure 8) are a visual alternative to the lambda value. In a completely random distribution (lambda = 1), the lines follow the 1:1 diagonal. If you found a few significant loci, but the rest of the distribution is random, the line follows the 1:1 diagonal, except near the end where it will rise up. That is the result you are hoping for. If the line is consistently above the 1:1 diagonal, the p-values are too significant overall and there appears to be a form of population stratification in your dataset. One way to correct this would be to perform a genomic-control correction with the lambda value - but you'd first want to check where this stratification is coming from and see if you can correct it that way. Frequently, such oversignificance can be filtered out by removing variants with low AF values or imputation quality. The M**anhattan plot** (figure 9) gives you a quick overview of whether there are significant findings present.

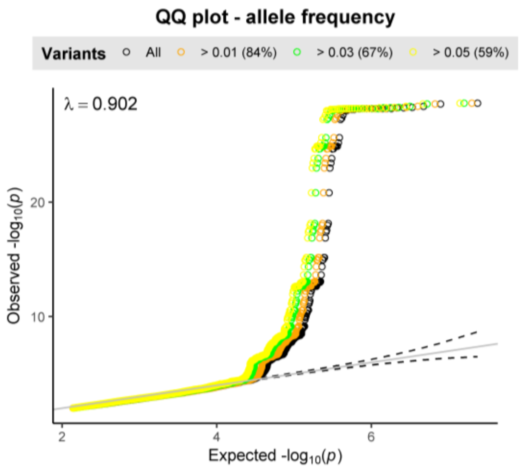

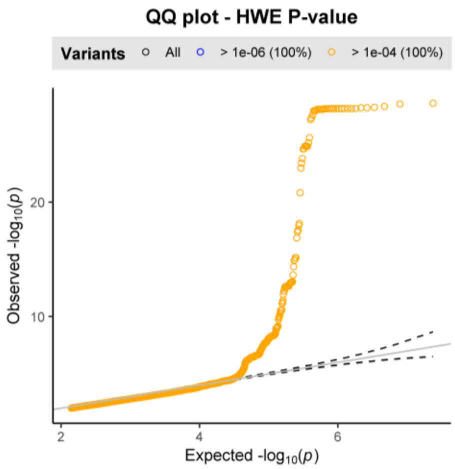

Insufficient data for "call rate" variable QQ-plot

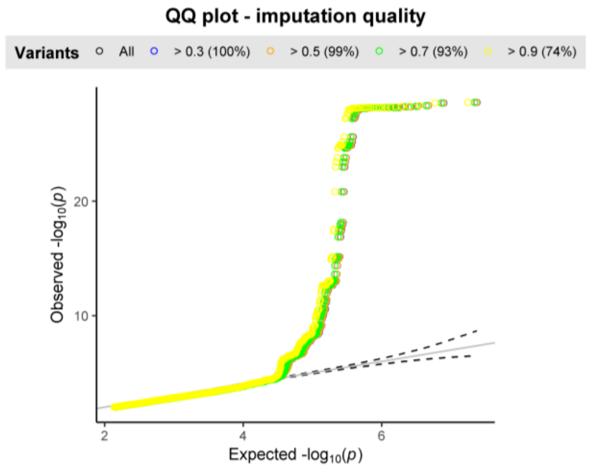

Figure 8: Sample QQ plots

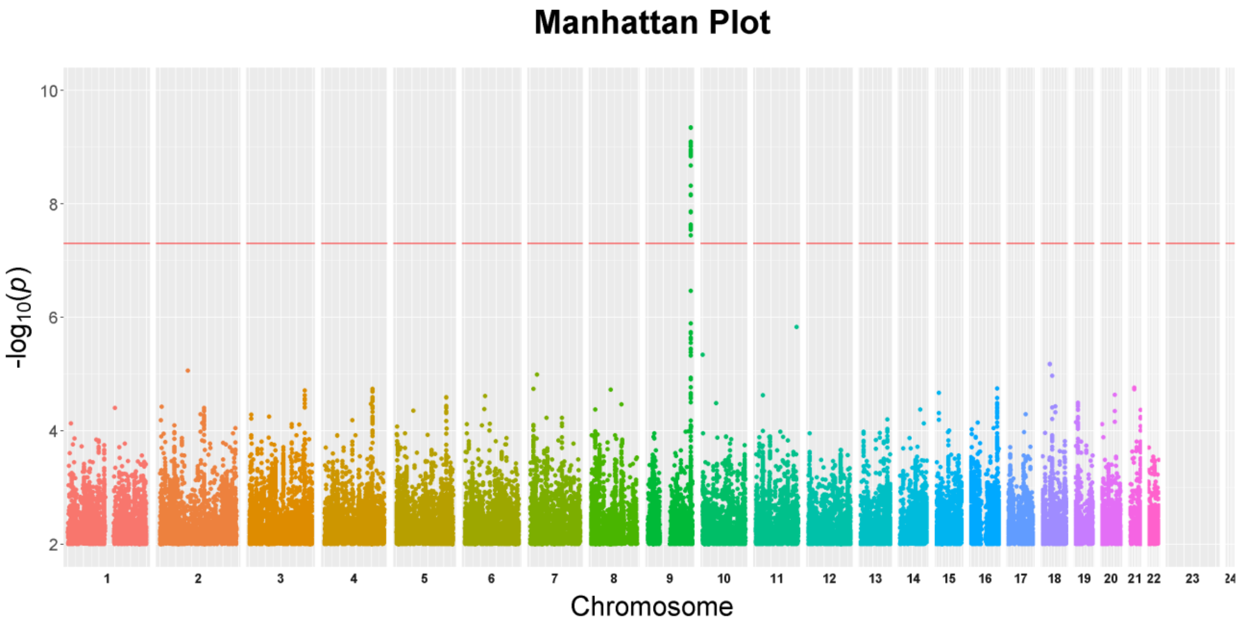

Figure 9: Sample Manhattan plot

Finally, GWASinspector may (depending on the input file) produce a series of .txt files (check here). These are lists of duplicated markers, markers whose alleles did not match with the reference, etc. They are intended to show the user what kind of data were excluded. Looking at them may give a hint as to what went wrong. Note that the files are not complete; usually only the first 100 entries are saved in order to save disk space and time.
