# Supplementary material for: GWASinspector: comprehensive quality control of genome-wide association study results
Source: Bioinformatics. 2021 Jan 8;37(1):129–30. doi: 10.1093/bioinformatics/btaa1084 (PMC8034536; doi:10.1093/bioinformatics/btaa1084)
Supplement: btaa1084_Supplementary_Data [file btaa1084_supplementary_data.zip › QC_sample_report.html]

Quality Check Report


# Quality Check Report

### GWASinspector package

---

QC start time: **Aug 16 2020 - 09:35:19 AM**

QC end time: **Aug 16 2020 - 09:56:08 AM**

Script version: **1.4.8**

System information : **R version 3.6.1 - 64bit**

### User preferences

Alterative header file: **alt\_headers.txt**

Allele frequency standard reference dataset: **1000G\_phase3\_GRCh37.sqlite**

Effect-size reference dataset: **effect-size\_reference.rds**

#### Filter values for selecting High-Quality (HQ) variants

|  | Value |
| --- | --- |
| Allele frequency | 0.05 |
| HWE p-value | 0.000001 |
| Call-rate | Not included |
| Imputation quality | 0.3 |

### Input File Description

Input file: **sample\_B.txt.gz**

Input file line count (including header): **15,790,126**

Input file ends with a new line: **TRUE**

#### Column names

|  |  |  |  |  |  |  |  |  |  |  |  |  |  |  |  |  |  |  |  |  |
| --- | --- | --- | --- | --- | --- | --- | --- | --- | --- | --- | --- | --- | --- | --- | --- | --- | --- | --- | --- | --- |
| Original | chr | position | STRAND | coded\_all | noncoded\_all | SE | Pvalue | AF\_coded\_all | HWE | information | imputed | n\_total | beta | RSID | av\_max\_posterior\_call | IQ | ALL\_AA | ALL\_AB | ALL\_BB | uniqsnpid |
| Renamed | CHR | POSITION | STRAND | EFFECT\_ALL | OTHER\_ALL | STDERR | PVALUE | EFF\_ALL\_FREQ | HWE\_PVAL | IMP\_QUALITY | IMPUTED | N\_TOTAL | EFFECT | RSID | AV\_MAX\_POSTERIOR\_CALL | IQ | ALL\_AA | ALL\_AB | ALL\_BB | UNIQSNPID |

*Header values are translated according to ‘header\_translations’ file.*

- missing columns = **MARKER | CALLRATE**

#### Column report

|  | NA values | Invalid values | Uncertain values |
| --- | --- | --- | --- |
| CHR | 0 | 0 \*\* |  |
| POSITION | 0 | 0 \* † |  |
| EFFECT\_ALL | 0 | 0 |  |
| OTHER\_ALL | 0 | 0 |  |
| EFFECT | 193118 |  |  |
| STDERR | 0 | 0 \* | 0 † |
| EFF\_ALL\_FREQ | 0 | 0 \* § | 0 ‡ |
| HWE\_PVAL | 0 | 0 \* † § | 0 ‡ |
| PVALUE | 0 | 0 \* † § | 0 ‡ |
| IMPUTED | 0 | 0 |  |
| IMP\_QUALITY | 0 | 0 ¶ |  |
| MARKER | 0 |  |  |
| N\_TOTAL | 0 | 0 \* † |  |
| STRAND | 0 | 0 |  |
| CALLRATE | 0 | 0 \* § | 0 ‡ |
|  |
| --- |
| Note: |
|  |
| \* < 0 |
| † = 0 |
| ‡ = -1 |
| § > 1 |
| ¶ valid range is between -0.5 and 1.5 |
| \*\* valid range is between 1 and 26 |

#### Variant processing

|  | Count |
| --- | --- |
| Input variant count | 15,790,125 |
| Missing crucial variable | 193,118 (1.2%) |
| Duplicated variants | 60 (0.00038%) |
| Variant count after step 1 \* | 15,596,947 (98.8%) |
| Monomorphic variants | 134,130 (0.85%) |
| Variant count after step 2 † | 15,462,817 (97.9%) |
| Variant count after step 3 ‡ | 15,432,412 (97.7%) |
|  |
| --- |
| Note: |
|  |
| \* step1: removing variants with missing crucial values and duplicated lines. |
| † step2: removing monomorphic variants and specified chromosomes. |
| ‡ step3: removing mismatched, ambiguous and multi-allelic variants that could not be verified. |

All further reports are based on variants after step3 (which will be saved as output file).

### Description of variants

Negative strand variants : **0**

Allele frequency = 0 : **132984**

Allele frequency = 1 : **1146**

|  | Count |
| --- | --- |
| High Quality variants | 6,089,688 (39%) |
| Low Quality variants | 9,342,724 (61%) |
| Palindromic variants\* | 2,138,677 (14%) |
| Non-Palindromic variants | 13,293,735 (86%) |
| Palindromic variants with high allele frequency difference (> 0.15) | 675 (0.032%) |
| Non-palindromic variants with high allele frequency difference (> 0.15) | 6,926 (0.052%) |
| Palindromic variants with opposite allele frequency “compared to the reference” (> 0.65 for the input file and < 0.35 for the reference, or vice versa) | 5 (0.00023%) |
|  |
| --- |
| Note: |
|  |
| \* Palindromic variants are variants with strand-independent allele-configurations (A/T and C/G) |

  
 Imputation status

|  | Count |
| --- | --- |
| imputed | 15432412 |

Variant count for each chromosome

|  |  |  |  |  |  |  |  |  |  |  |  |  |  |  |  |  |  |  |  |  |  |  |
| --- | --- | --- | --- | --- | --- | --- | --- | --- | --- | --- | --- | --- | --- | --- | --- | --- | --- | --- | --- | --- | --- | --- |
| Chromosome | 1 | 2 | 3 | 4 | 5 | 6 | 7 | 8 | 9 | 10 | 11 | 12 | 13 | 14 | 15 | 16 | 17 | 18 | 19 | 20 | 21 | 22 |
| Count | 1,193,483 | 1,306,953 | 1,112,319 | 1,116,394 | 988,062 | 1,020,854 | 905,867 | 854,664 | 668,111 | 776,022 | 761,915 | 740,070 | 568,816 | 503,327 | 444,229 | 483,312 | 422,482 | 441,602 | 354,374 | 345,126 | 210,124 | 214,306 |

- Missing chromosome(s) number **23**

### Result from matching variants with reference datasets

#### Variant types after matching with standard reference datasets

|  | count |
| --- | --- |
| Bi-allelic SNP | 12,783,423 |
| Multi-allelic SNP | 1,061,172 |
| SNPs not found in standard reference dataset | 37,082 |
| Bi-allelic INDEL | 1,297,088 |
| Multi-allelic INDEL | 94,936 |
| INDELs not found in standard reference dataset | 158,711 |

#### Result of matching with standard reference dataset

|  | Count |
| --- | --- |
| Verified variants | 15,236,619 (98.5%) |
| Not-found variants | 195,793 (1.27%) |
| Flipped variants | 1,716 (0.011%) |
| Switched variants | 0 (0%) |
| Allele frequency correlation |  |
| r (all variants) | 0.997 |
| r (palindromic) | 0.997 |
| r (non-palindromic) | 0.997 |
| r (INDEL) | 0.99 |


---

### QC summary statistics

#### P-value correlation (observed vs expected)

**Note:** Only variants with a valid P-value are used for P-value correlation calculation.

|  | Value |
| --- | --- |
| Included variants | 15,432,412 (100%) |
| r | 1 |


---

#### Distribution statistics [1]

|  | Value |
| --- | --- |
| Skewness | 0.656 |
| Skewness (HQ) | -0.19 |
| Kurtosis | 14.2 |
| Kurtosis (HQ) | 133 |
| Visscher’s stat | 3.41 |
| Visscher’s stat (HQ) | 3.41 |
| Lambda - total | 0.994 |
| Lambda - genotyped | NA |
| Lambda - imputed | 0.994 |
| Sample Size (max) | 2489 |
| Fixed HWE P-value | No |
| Fixed Imputation Quality | No |
| Fixed Call Rate | NA |
| Fixed Sample Size | YES (2489) |
|  |
| --- |
| Note: |
|  |
| \* **HQ** = High-Quality variants |

---

#### Distribution statistics [2]

##### All variants

|  | Min. | 1st Qu. | Median | Mean | 3rd Qu. | Max. |
| --- | --- | --- | --- | --- | --- | --- |
| PVALUE | 1.441e-14 | 0.25 | 0.5012 | 0.5003 | 0.7509 | 1 |
| HWE\_PVAL | 1.313e-06 | 0.5675 | 1 | 0.7719 | 1 | 1 |
| EFF\_ALL\_FREQ | 0.001 | 0.004 | 0.033 | 0.1698 | 0.247 | 0.999 |
| IMP\_QUALITY | 0.1 | 0.6069 | 0.8562 | 0.7467 | 0.9553 | 1 |
| BETA | -2.474 | -0.02977 | -0.0001455 | 0.00266 | 0.02931 | 3.068 |
| STDERR | 0.01258 | 0.01829 | 0.04799 | 0.1149 | 0.1842 | 0.4623 |

---

##### HQ variants

|  | Min. | 1st Qu. | Median | Mean | 3rd Qu. | Max. |
| --- | --- | --- | --- | --- | --- | --- |
| PVALUE | 1.305e-07 | 0.2495 | 0.5008 | 0.5003 | 0.7516 | 1 |
| HWE\_PVAL | 1.313e-06 | 0.3331 | 0.5973 | 0.5739 | 0.8324 | 1 |
| EFF\_ALL\_FREQ | 0.05 | 0.128 | 0.27 | 0.3397 | 0.509 | 0.95 |
| IMP\_QUALITY | 0.3 | 0.7198 | 0.8854 | 0.8169 | 0.9623 | 1 |
| BETA | -1.353 | -0.01234 | -4.943e-05 | 2.765e-05 | 0.01235 | 1.237 |
| STDERR | 0.01258 | 0.01456 | 0.01725 | 0.02066 | 0.02278 | 0.4623 |

#### Effect allele distribution in SNP variants

|  |  |  |  |  |
| --- | --- | --- | --- | --- |
| Allele | A | C | G | T |
| Count (input file) | 3847186 | 3090233 | 3097190 | 3847068 |
| Count (post-matching) | 3847186 | 3090233 | 3097190 | 3847068 |

#### Other allele distribution in SNP variants

|  |  |  |  |  |
| --- | --- | --- | --- | --- |
| Allele | A | C | G | T |
| Count (input file) | 2991870 | 3951860 | 3950215 | 2987732 |
| Count (post-matching) | 2991870 | 3951860 | 3950215 | 2987732 |

### Effect-size correlation

|  | r\* | r\*\* |
| --- | --- | --- |
| P-value < 0.001 | 0.6118 (3161) | 0.9788 (78) |
| P-value < 0.0001 | 0.7846 (762) | 0.9728 (29) |
| P-value < 0.00001 | 0.9256 (304) | 0.9683 (11) |
| P-value < 0.000001 | 0.9407 (181) | 0.9528(7) |
|  |
| --- |
| Note: |
| \* = Data is presented as r(N). Variants were filtered on reference data P-values. |
| \*\* = Data is presented as r(N). Variants were filtered on input result file P-values. |

### Plots


---


---
